# Transcriptomic but not genomic variability confers phenotype of breast cancer stem cells

**DOI:** 10.1186/s40880-018-0326-8

**Published:** 2018-09-19

**Authors:** Mengying Tong, Ziqian Deng, Mengying Yang, Chang Xu, Xiaolong Zhang, Qingzheng Zhang, Yuwei Liao, Xiaodi Deng, Dekang Lv, Xuehong Zhang, Yu Zhang, Peiying Li, Luyao Song, Bicheng Wang, Aisha Al-Dherasi, Zhiguang Li, Quentin Liu

**Affiliations:** 1 Center of Genome and Personalized Medicine, Institute of Cancer Stem Cell, Dalian Medical University, Dalian, 116044 Liaoning P. R. China; 2 State Key Laboratory of Oncology in South China, Collaborative Innovation Center of Cancer Medicine, Sun Yat-sen University Cancer Center, Guangzhou, Guangdong 510060 P. R. China; 3 Cancer Center, Union Hospital, Tongji Medical College, Huazhong University of Science and Technology, Wuhan, Hubei 430022 P. R. China

**Keywords:** Breast cancer, Cancer stem cell, Genomics, Sequencing, Transcriptomics

## Abstract

**Background:**

Breast cancer stem cells (BCSCs) are considered responsible for cancer relapse and drug resistance. Understanding the identity of BCSCs may open new avenues in breast cancer therapy. Although several discoveries have been made on BCSC characterization, the factors critical to the origination of BCSCs are largely unclear. This study aimed to determine whether genomic mutations contribute to the acquisition of cancer stem-like phenotype and to investigate the genetic and transcriptional features of BCSCs.

**Methods:**

We detected potential BCSC phenotype-associated mutation hotspot regions by using whole-genome sequencing on parental cancer cells and derived serial-generation spheres in increasing order of BCSC frequency, and then performed target deep DNA sequencing at bulk-cell and single-cell levels. To identify the transcriptional program associated with BCSCs, bulk-cell and single-cell RNA sequencing was performed.

**Results:**

By using whole-genome sequencing of bulk cells, potential BCSC phenotype-associated mutation hotspot regions were detected. Validation by target deep DNA sequencing, at both bulk-cell and single-cell levels, revealed no genetic changes specifically associated with BCSC phenotype. Moreover, single-cell RNA sequencing showed profound transcriptomic variability in cancer cells at the single-cell level that predicted BCSC features. Notably, this transcriptomic variability was enriched during the transcription of 74 genes, revealed as BCSC markers. Breast cancer patients with a high risk of relapse exhibited higher expression levels of these BCSC markers than those with a low risk of relapse, thereby highlighting the clinical significance of predicting breast cancer prognosis with these BCSC markers.

**Conclusions:**

Transcriptomic variability, not genetic mutations, distinguishes BCSCs from non-BCSCs. The identified 74 BCSC markers have the potential of becoming novel targets for breast cancer therapy.

**Electronic supplementary material:**

The online version of this article (10.1186/s40880-018-0326-8) contains supplementary material, which is available to authorized users.

## Background

Traditional breast cancer therapies that target bulk cell populations often have substantial short-term effects, and the existence of breast cancer stem cells (BCSCs) is a major barrier for achieving curability. BCSCs are cells that have the ability to self-renew, and they are considered responsible for key aspects of tumors, such as tumor initiation, progression, and drug resistance [1–5]. Therefore, approaches targeting BCSCs have important clinical implications [6]. Although several discoveries have been made on BCSC characterization [7–9], the origin of BCSCs is still unclear. Currently, two models are usually proposed to explain BCSCs. The first model [10, 11] is clonal evolution, in which tumor cells progressively accumulate mutations, some of which confer the ability of self-renewal and allow tumor cells with these mutations to become BCSCs and out-compete other tumor cells. This model is a canonical hardwired BCSC hierarchy, and BCSC dedication is largely defined by intrinsic genetic properties. In the second model [12, 13], BCSCs do not necessarily acquire mutations and are instructed by dedicated niche signals following competition dynamics. Breast cancer cells can be reprogrammed into BCSCs through plasticity in tumor microenvironment.

To determine the factors critical to the origination of BCSCs, we assumed that genetic mutations contributes to the acquisition of cancer stem-like phenotypes, e.g., BCSCs specifically carry heritable genetic changes. We then tested this hypothesis using next-generation sequencing (NGS) analysis, including whole-genome sequencing (WGS), target deep DNA sequencing, and transcriptome sequencing at both bulk-cell and single-cell levels.

## Materials and methods

### Cell culture

The human breast cancer cell line MDA-MB-231 was obtained from the American Type Culture Collection (ATCC). The cell line was authenticated at ATCC before purchase by the standard short tandem repeat DNA typing and cultured in its standard medium as recommended by ATCC.

### Sphere formation assay

The single-cell suspension was obtained by trypsinization. Clumped cells were excluded with a 40-μm sieve. Single cells were plated in ultra-low attachment 6-well plates (Corning, NY, USA) at a low density (1000 cells/well). The cells were maintained in serum-free Dulbecco’s modified eagle medium/nutrient mixture F12 (DMEM/F12, Gibco, Waltham, MA, USA) supplemented with B27 (Invitrogen, Waltham, MA, USA), 20 ng/mL epidermal growth factor (EGF, Sigma, Darmstadt, Germany), and 20 ng/mL basic fibroblast growth factor (bFGF, BD Biosciences, Franklin Lakes, NJ, USA) for 7–10 days. For sphere passage, the spheres were collected by centrifugation (1000 rpm, 5 min), dissociated with trypsin-ethylene diamine tetraacetic acid (EDTA), and mechanically dispersed. The resulting single cells were then centrifuged (1000 rpm, 5 min) to remove the enzyme and re-suspended in serum-free medium. The spheres were passed every 7–10 days, and only spheres bigger than 50 μm in diameter were included in the analyses.

### Serial sphere formation assay

The single-cell suspension of MDA-MB-231 cells was obtained by trypsinization. Cells were seeded in an ultra-low attachment 96-well plate (1 cell/well). The cells were maintained in serum-free DMEM/F12 supplemented with B27, and 20 ng/mL EGF, 20 ng/mL bFGF. Only wells that initially contained a single cell were used for subsequent studies. For sphere passage, the single cell-derived sphere was sucked up by a micro-pipette, dissociated with a small amount of trypsin-EDTA, and mechanically dispersed. The resulting single cells were then re-suspended in serum-free medium. The spheres were passed every 7–10 days, and only spheres bigger than 50 μm in diameter were included in the analyses.

### ALDEFLUOR assay by fluorescence activated cell sorting (FACS)

The ALDEFLUOR kit (STEMCELL, Vancouver, British Columbia, Canada, Cat. 01700) was used for isolating the cell population with high aldehyde dehydrogenase (ALDH) enzymatic activity. Cells were suspended in an ALDEFLUOR assay buffer containing ALDH substrate bodipy aminoacetaldehyde (BAAA, 1 mol/L per 1 × 10^6^ cells) and incubated for 45 min at 37 °C. As negative controls, for each example of cells, an aliquot was treated with 50 mmol/L diethylaminobenzaldehyde (DEAB), a specific ALDH inhibitor. The ALDH-positive subpopulation was isolated by FACS.

### Transwell invasion assay

Parental cells (MDA-MB-231) were obtained by trypsinization and resuspended in pure DMEM. Spheres bigger than 50 μm were obtained through a 40-μm cell strainer (Meilun Biotechnology, Dalian, Liaoning, China). Then the spheres were centrifuged (1000 rpm, 5 min), dissociated with trypsin-EDTA, and mechanically dispersed in pure DMEM. For every chamber, 30,000 cells were placed onto 1% matrigel (BD Biosciences)-coated membrane in the upper chamber (24-well insert, 8 μm, Corning, Cat. 3422). Medium with 10% fetal bovine serum (FBS, Gibco) was used as an attractant in the lower chamber. After being incubated for 24 h, cells that invaded through the membrane were fixed with 4% paraformaldehyde and stained with 0.1% crystal violet. The stained cell images were captured under a microscope (Olympus, Tokyo, Japan), and cells were counted for five random fields at ×10 magnification. Results are presented as mean ± standard deviation from at least three independent experiments.

### RNA extraction, reverse transcription-PCR, and quantitative real-time PCR

Total RNA was extracted by using TRIzol reagent (Life Technologies, Waltham, MA, USA). cDNA was generated by reverse transcription-PCR using EasyScript One-Step gDNA Removal and cDNA Synthesis SuperMix Kit (TransGen Biotech, Beijing, China) according to the manufacturer’s instructions. Quantitative real-time polymerase chain reaction (qRT-PCR) was performed by using the chamQ Universal SYBR qPCR Master Mix (Vazyme, Najing, Jiangsu, China) in a MX3000p cycler (Stratagene, La Jolla, CA, USA). Changes of mRNA levels were detected by the 2^−ΔΔCT^ method using Actin for internal crossing normalization. Detailed primer sequences for qRT-PCR are listed in Additional file 1: Table S1.

### Western blot analysis

Samples were lysed on ice in RIPA buffer (50 mmol/L Tris [pH 8.0], 150 mmol/L sodium chloride, 0.5% sodium deoxycholate, 0.1% sodium dodecyl sulfate, and 1% NP-40) supplemented with protease inhibitors [1 mmol/L Na_3_VO_4_, 1 μg/mL leupeptin, and 1 mmol/L phenylmethanesulfonyl fluoride (PMSF)]. The protein concentration was detected by the Coomassie brilliant blue dye method. In all, equal amounts of protein per lane were run in 10% sodium dodecyl sulfate-polyacrylamide electrophoresis gels and subsequently transferred to a nitrocellulose membrane (Millipore, Darmstadt, Germany) via submerged transfer. After blocking the membrane with 5% milk at room temperature for 1 h, the membrane was incubated overnight at 4 °C with various primary antibodies. After incubation with peroxidase-conjugated secondary antibodies (Thermo Scientific, Waltham, MA, USA) for 1 h at room temperature, the signals were visualized using an enhanced chemiluminescence western blot detection kit (K-12045-D50; Apgbio, Beijing, China) according to the manufacturer’s instructions. The blots were developed using the Bio-Rad Molecular Imager instrument (Bio-Rad, Berkeley, CA, USA). The information regarding the antibodies used are listed as follows: mouse anti-human monoclonal ACTB antibody (Proteintech, Chicago, IL, USA, 66009-1-Ig), rabbit anti-human monoclonal NANOG antibody (Abcam, Cambridge, England, ab109250), mouse anti-human monoclonal SOX2 antibody (Santa Cruz Biotechnology, Santa Cruz, CA, USA, sc-365823).

### Whole-genome sequencing and data processing in bulk cells

#### DNA extraction, library preparation, and sequencing

The genomic DNA of bulk cells (1 × 10^6^ cells) was extracted using the ALLPrep DNA/RNA Mini Kit (Qiagen, Hilden, Germany, Cat. 80204) according to the manufacturer’s manual. Quantified 50 ng genomic DNA was used to prepare the paired-end library using the TruePrep DNA library Prep Kit V2 for Illumina (Vazyme, Cat. TD-501). The quality and concentration of DNA fragments in the DNA libraries generated were assessed using High-Sensitivity Bioanalyzer (Agilent, Santa Clara, CA, USA). The prepared library was then subjected to Illumina HiSeqXten Sequencer (San Diego, CA, USA) with the paired-end 150 bp read option.

#### Reads mapping and variants calling

The Feb. 2009 human reference sequence (GRCh37) was used in this study, and it was produced by the Genome Reference Consortium. BWA MEM (version 0.7.12) was used to align all paired-end reads to the Hg 19 reference genome with default parameters. We performed base quality score recalibration and local realignment using the Genome Analysis Toolkit (GATK, version 3.6). The duplicated reads were marked using the function “MarkDuplicates” of Picard Tools (version 1.126) and then removed. Following this, variants were called using SAMtools mpileup with the following parameters: -Q 30 –q 10. The variants [single nucleotide variant (SNV) and indel] were identified by VarScan (version 2.3.7) mpileup2cns with the following parameters: --min-coverage 2 --min-reads2 1 --variants 1 --*P* value 0.05 --min-var-freq 0.1.

#### Variant filtering

In each sample [the parental cells (2D) and derived spheres of the first generation (SP1) and fourth generation (SP4)], putative SNVs and indels were filtered with the following criteria: (1) calls falling on the mitochondria genome, Y chromosome, unknown chromosome, genomic SuperDups, and RepeatMasker regions (available on the download page at the University of California Santa Cruz website (http://www.genome.ucsc.edu/) were removed and (2) depth range from 10 to 200. Finally, we obtained 1,628,063 variant sites, including SNV and indel, existing in at least one sample.

#### Further variant selection for hotspot calling

We assumed that sample 2D possess the lowest proportion of BCSCs, while SP4 the highest; then, the proportion of the cell population carrying variants were increased from 2D to SP4, which was quantified by variant allele frequency (VAF). Thus, to select significantly increased sites based on VAF between 2D and SP4, we performed the Fisher’s exact test on the read counts supporting the reference and variation in each site. A total of 30,797 sites were considered significantly increased from 2D to SP4 and were selected for calling hotspots following the two conditions: *P* value less than 0.1 and the VAF of 2D less than the VAF of SP4.

### Target deep DNA sequencing and data processing in bulk cells

#### Amplicon primer design

We selected 54 hotspots for the multi-PCR target validation design. ION AmpliSeq Designer (http://www.ampliseq.com) was able to successfully design amplicon primers for 97% of the targets. For hotspots with a length less than 500 bp, amplicon primers were designed to cover the whole regions, otherwise, amplicon primers were designed covering the SNV sites in the hotspots. According to this principle, we designed 128 amplicons to target these 54 hotspots (Additional file 2: Table S2).

#### Library preparation and sequencing for amplicons

The detailed protocol of target deep DNA sequencing was as follows:

A. Multiplex PCR amplification: The 128 amplicons were amplified by multiplex PCR on Veriti 96-well Thermal Cycler (Applied Biosystems, Waltham, MA, USA), which was performed using 30 ng genomic DNA, 15 μL Primer mix/pool (2 pools in total), 10 μL Q5 reaction buffer (NEB, Ipswich, MA, USA, Cat. B9027S), 10 μL Q5 high GC enhancer (NEB, Cat. B9028A), 1.5 μL dNTPs mix (NEB, Cat. N0447S), 0.5 μL Q5 high-fidelity DNA polymerase (NEB, Cat. M0491L), and ddH_2_O to make the final reaction volume to 50 μL. The reaction system was incubated initially at 98 °C for 30 s. Fifteen cycles of PCR were performed at 98 °C for 10 s and 62 °C for 4 min. Then, the reaction was held at 4 °C.
B. Column purification of PCR product: All PCR products were purified using DNA Purification Kit (TIANGEN, Beijing, China, Cat. DP214-03).
C. End repair and A-tailing of DNA fragments: The mixture of 37.5 μL DNA, 5 μL Cut Smart (NEB, Cat. B7204S), 5 μL Adenosine 5′-Triphosphate (NEB, Cat. P0756L), 0.5 μL of 100 mmol/L dATP solution (NEB, Cat. N0440S), 1 μL T4 Polynucleotide Kinase (NEB, Cat. M0201L) and 1 μL 5 units Klenow exo-DNA polymerase (NEB, Cat. M0212L) was incubated at 37 °C for 1 h on Veriti 96-well Thermal Cycler.
D. Column purification: The PCR products were purified with Universal DNA Purification Kit (TIANGEN, Cat. DP214-03).
E. Adapter ligation: The mixture of 25 μL A-tailed DNA, 1 μL of 50 μmol/L multiplexing adapter, 3 μL of 10× T4 DNA ligase buffer (NEB, Cat. B0202), and 1 μL of 400 units/μL T4 DNA ligase (NEB, Cat. M0202L) was used in adapter ligation step followed by incubation at 16 °C overnight.
F. Ampure cleanup of adapter-ligated reaction: We added 1 × volume (30 μL) of Agencourt AMPure XP DNA beads (BECKMAN, Brea, CA, USA, Cat. 15604000) and incubated at room temperature for 5–10 min and then placed on magnetic stand. We discarded the supernatant which contained primer dimers. Beads were washed twice with 200 μL of 80% ethanol for 30 s at room temperature and dried at room temperature for 5–10 min. Then, 25 μL Buffer EB was added to the beads, mixed up and down for ten times, incubated for 2 min at room temperature, and put on magnetic stand at room temperature for about 5 min. After that, 22 μL of supernatant was transferred to a new PCR tube.
G. PCR amplification: PCR enrichment was conducted by using 22 μL Adapter-ligated DNA, 25 μL of 2 × NEB Next high-fidelity PCR master buffer (NEB, Cat. M0541L), 1.5 μL of 10 μmol/L MUP primer, 1.5 μL of 10 μmol/L barcode primer. The reaction was incubated initially at 98 °C for 3 min. Fifteen cycles of PCR were performed at 98 °C for 20 s, 65 °C for 15 s, and 72 °C for 20 s. The reaction was then held at 4 °C.
H. Extraction and purification of the final library: electrophoresis was conducted in agarose gel with the PCR products from the last step and then extracting and purifying DNA from agarose gel using the gel extraction kit (TIANGEN, Cat. DP214-03).
I. Quality control and sequencing of the final library: The quality and concentration of DNA fragments in the DNA libraries generated were assessed using High-Sensitivity Bioanalyzer. The prepared library was then subjected to Illumina HiSeqXten with the paired-end 150 bp read option.

#### Data processing

The sequence of primer regions was first trimmed off from the fastq data by PrimerTrim. (available at http://github.com/DMU-lilab). BWA MEM (version 0.7.12) was then used to align primer-removed reads to the Hg 19 reference genome with default parameters. We removed secondary alignments and alternative hits by SAMtools with the following parameters: x XA –F 0x100. Then, variants were called using SAMtools mpileup with the following parameters: -Q 30 -q 10. The variants (SNV and indel) were identified by VarScan (version 2.3.7) mpileup2cns with the following parameters: --min-coverage 60 --min-reads2 1 --variants 1 --*P* value 0.05 --min-var-freq 0.1.

### Single-cell target deep DNA sequencing

#### Characterization of the cancer hotspot mutation (CHM) panel and amplicon design

The targeted genes and mutations are listed in Additional file 3: Table S3, including the whole exonic region of 48 cancer hotspot genes (from 50 cancer hotspot genes identified by the Mayo Clinic) and 1513 mutations. Of the 1513 mutations, 224 were identified from The Cancer Genome Atlas (TCGA, https://cancergenome.nih.gov/) data, and 1286 were identified from the Catalogue Of Somatic Mutations In Cancer (COSMIC, https://cancer.sanger.ac.uk/cosmic/) data, and 3 were identified from both databases. ION AmpliSeq Designer (Thermo Fisher, http://www.ampliseq.com) was used to design 128 amplicons covering the WGS hotspot mutation (WHM) panel (Additional file 2: Table S2) and 3124 amplicons covering the hotspots in the CHM panel (Additional file 4: Table S4).

#### Single-cell isolation, genomic DNA extraction, and multiple displacement amplification (MDA)

Single cells or single spheres were sucked up by a micro-pipette. Whole genome amplification (WGA) was performed to these cells using the Discover-sc Single Cell Kit (Vazyme, Cat. N601-01) according to the manufacturer’s manual, and a reaction of human tissue genomic DNA was marked as a positive control. The amplified DNA products were then stored at − 20 °C.

#### Quantification and genome-integrity assessment of WGA products

The DNA concentration of the WGA products was measured using the Qubit Quantitation platform (Life Technologies, Invitrogen, Waltham, MA, USA). Ten housekeeping genes located on different chromosomes were selected to check the coverage of amplified products. WGA products of best performance in relation to housekeeping PCR (> 8/10) and Qubit assays (> 60 ng/μL) were selected for downstream experiments. All of the above steps were performed with a sample of genomic DNA from human tissue as a positive control.

#### Library preparation and sequencing for amplicons

Target regions were amplified by multiplex PCR in WGA products. Choosing the correct number of cycles for the multiplex PCR is critical based on the starting amount and coverage of WGA products. The quality and concentration of the DNA libraries generated was assessed using High-Sensitivity Bioanalyzer. The prepared target libraries were then subjected to Illumina HiseqXten with the paired-end 150 bp read option.

### Single-cell RNA sequencing (scRNA-seq)

#### Generation of scRNA-seq libraries

The generation of single-cell cDNA libraries was implemented by the Discover-sc WTA Kit (Vazyme, Cat. N711-01) according to the manufacturer’s manual for single cell-derived spheres and single cells. Quantified 1 ng amplified cDNA was then used to prepare the paired-end library using TruePrep DNA library Prep Kit V2 for Illumina (Vazyme, Cat. TD-503). The quality and concentration of DNA fragments in the cDNA libraries generated was assessed using High-Sensitivity Bioanalyzer. Massively parallel RNA sequencing (RNA-seq) was performed on the Illumina HiSeqXten platform with paired-end 150-bp read-length by Berry Genomic Corporation (Beijing, China).

#### Data analysis

TopHat2 was used to align reads according to the University of California Santa Cruz hg19 reference genome, and the corresponding gene annotation format file from GENCODE was fed to the TopHat2 for defining transcript coordinates. Gene-level expression abundance (fragments per kilobase of exon per million fragments mapped) and the results of differential gene expression analysis were obtained from the Cufflinks package. By the comparison between BCSCs and non-BCSCs, we identified the differentially expressed gene set according to the fold change (FC) and false discovery rate (FDR). Genes with FDR < 0.05 and log2-transformed FC > 1 were considered to be highly expressed in BCSCs.

### Gene set enrichment analysis

The gene expression dataset containing gene symbols and gene expression values of 8 single-cell RNA-seq samples were submitted to gene set enrichment analysis (GSEA) (version v2.2.1) software [14] according to the GSEA user guide. GSEA was performed with the gene set of MSigDB: Gene Ontology Biological Process. The nominal *P* value < 0.01 and FDR < 0.25 were used to investigate significantly enriched gene sets.

### Kaplan–Meier (KM) survival analysis

To investigate the association between BCSC highly expressed genes and patient survival, we evaluated the relapse-free survival after surgery in all patients available in the Kaplan–Meier plotter online database [15] (http://kmplot.com/analysis/index.php?p=background). A user-selected probe set was chosen, and patients were grouped according to the optimized cut-off.

### Interaction analysis in the STRING database

A newly identified BCSC marker gene set was submitted to the STRING database [16] (https://string-db.org/cgi) to identify associated genes and pathways. Interactions including curated databases and experimentally determined gene neighborhoods, gene fusions, gene co-occurrence, text mining, co-expression, and protein homology were investigated.

### Survival analysis in pan-cancer

The table of survival *z* scores collapsed by cancer/cancer subtype was downloaded from the PREdiction of Clinical Outcomes from Genomic Profiles (PRECOG) database (https://precog.stanford.edu/index.php) [17]. For the *z* scores of the BCSC marker gene set in pan-cancer, we made a hierarchical clustering analysis in R (version 3.3.2) using the hclust function (https://stat.ethz.ch/R-manual/R-devel/library/stats/html/hclust.html).

### Biomarker validation by SurvExpress

Nine breast cancer relapse datasets were analyzed for the BCSC marker gene set in the SurvExpress online database [18] (http://bioinformatica.mty.itesm.mx:8080/Biomatec/SurvivaX.jsp). A Cox regression model was used to generate 2 risk groups by splitting the samples at the median after ranking by their prognostic index, which were estimated using beta coefficients multiplied by gene expression values. The box plot obtained as the results of SurvExpress visualized the expression levels of each gene in the risk groups generated. The *P* value was obtained from a *t* test for two groups.

### Analysis of differential gene expression between cancer and normal tissues from the TCGA dataset

Gene expression data of 38 cancer types were downloaded from FireBrowse (http://firebrowse.org). To ensure sufficient statistical power, the number of either normal or cancer samples was at least 5, and 22 of 38 cancer types meet the requirement and were used for the following analysis. Differential gene expression between normal and cancer samples were evaluated by *t* test.

### Statistical analyses

#### Hotspot calling

After potential BCSC-associated SNV sites were identified by WGS in bulk cells, a statistical model was established to call hotspot regions where variants (30,797 variants in all) were densely distributed. When the mutations in a given length of DNA were considered as Poisson distributed, and the distance between two adjacent mutations followed an exponential distribution (Additional file 7: Fig. S1a). Then, the probability of a given distance could be calculated as follows:

$$P\left( {\text{x}} \right) = 1 - e^{{ -\uplambda{\text{x}}}} \left( {\uplambda = \frac{1}{{\bar{x}}}, {\text{x}} > 0} \right)$$

where x refers to the distance between two adjacent SNV sites and $\bar{x}$ refers to the average distance of all two adjacent SNV sites in the genome.

Thereafter, hotspot regions were detected using Run-length encoding (RLE) algorithm (Additional file 7: Fig. S1b). Hotspots with length longer than 100,000 bp were removed, and 54 hotspots were obtained (Additional file 5: Table S5). Specifically, the median length of the hotspots was 318 bp, and most hotspots overlapped with intronic and intergenic regions (Additional file 7: Fig. S1c and S1d). Subsequently, we calculated the *P* value evaluating the significance of the hotspot regions as follows:

$$P\left( {X \ge m} \right) = 1 - P\left( {X < m} \right) = 1 - \mathop \sum \limits_{i = 0}^{m - 1} \frac{{\left( {\begin{array}{*{20}c} b \\ i \\ \end{array} } \right) \times \left( {\begin{array}{*{20}c} {a - b} \\ {n + m - i} \\ \end{array} } \right)}}{{\left( {\begin{array}{*{20}c} a \\ {n + m} \\ \end{array} } \right)}}$$

where a stands for the total number of distances of two adjacent SNV sites, b stands for the number of distances whose exponential distribution *P* value (*P*_exp_) < 0.01 (in our case a = 30,774, b = 4003), n stands for the number of distances allowed within a hotspot whose *P*_exp_ ≥ 0.01, and m stands for the number of distances whose *P*_exp_ < 0.01. X is the random variable representing the number of distances with *P*_exp_ < 0.01 in a region. Under the minimum requirement (*m* = 5, *n* = 1) of our hotspot-calling algorithm, *P* was equal to 1.988 × 10^−4^.

#### Computation of the genetic distance between every two samples (single cells)

At a given position, the genetic distance between C1 and C2 was exemplified as follows. We defined C1 = (C_1_W_A_, C_1_W_T_, C_1_W_G_, C_1_W_C_) and C2 = (C_2_W_A_, C_2_W_T_, C_2_W_G_, C_2_W_C_), where C_1_W_A_ refers to the weight (i.e., proportion) of read counts supporting base “A” of sample C1, and C_2_W_T_ refers to the weight of read counts supporting base “T” of sample C2, as an analogy). Then, the genetic distance (d) between C1 and C2 was calculated by the Pythagorean formula:

$$d\left( {C1,C2} \right) = \sqrt {\left( {\mathop \sum \limits_{n = A,T,G,C} \left( {C_{1} W_{n} - C_{2} W_{n} } \right)} \right)^{2} }$$

#### Analysis of base-position differences between BCSCs and non-BCSCs (single cells)

- Step 1. Acquiring for binary alignment (BAM) files.
  BAM files were generated using the pipeline identical to the target deep DNA sequencing of bulk cells, as described under “Data processing” in the subsection “Target deep DNA sequencing and data processing in bulk cells”.
- Step 2. Count data.
  Nucleotides were counted from recalibrated BAM files using Rsamtools (http://bioconductor.org/packages/release/bioc/html/Rsamtools.html). Only positions in the target regions and covered by all samples were kept for further analysis. Here, the following was denoted for the considered position:
  Total count (TC) = sum of A, T, C, and G counts
  Major count (MC) = the highest nucleotide count
  Background count (BC) = TC − MC
  Subscript *c* = BCSCs
  Subscript *n* = non-BCSCs
- Step 3. Position error rate [19] (PER) of non-BCSCs (the two single cells).
  At each position, we estimated the PER of non-BCSCs as follows:
  $$PER_{n} = \frac{{\sum BC_{n} }}{{\sum TC_{n} }}$$
- Step 4. Binomial analysis of BCSCs (the three spheres).
  At each base position, we calculated the probability of BC_c_ (PBC) from a binomial distribution with the parameter PER (corrected). PBC represents the statistical probability to observe the specific number of background allele at a position. PER was obtained as in Step 3 from the two non-BCSC single cells. The following is the PBC calculation formula:
  $$P = {\text{binom}} . {\text{test}} \left( {\frac{{\sum BC_{c} }}{3},\frac{{\sum TC_{c} }}{3},PER_{n} + c\sqrt {\frac{{PER_{n} }}{{TC_{n} }}} ,{\text{alternative}} = {\text{"greater"}} }\right)$$
  where c represents the quantile of order 1-alpha of the standard Gaussian, with c equaling to 1.64 (quantile of order 95% for the Gaussian) Binomial test was the R function to obtain an exact test of a simple null hypothesis about the probability of success in a Bernoulli experiment (https://www.rdocumentation.org/packages/stats/versions/3.4.3/topics/binom.test).
- Step 5. Permutation.
  The case group denoted the group of 3 BCSCs versus 2 non-BCSCs, and the other 9 (i.e., *C*_5_^2^ − 1) random arrangements were defined as permutation groups (Additional file 6: Table S6). The general approach for calculating the *P* values of permutation groups was the same as in Step 4.
- Step 6. Case-permutation ratio (CPR).
  After the computation of the *P* value of each position in each group, we counted the number of positions (NP) with *P* value less than the specific *P* value threshold (ranging from 0 to 0.1) in each group. To assess the difference in NP between the case group and each permutation group, we defined CPR as follows:
  $${\text{CPR}} = \frac{{NP_{case} }}{{NP_{permutation} + NP_{case} }}$$

#### Pearson correlation analysis, Fisher’s exact test, and Student’s t test

Pearson correlation analysis and Fisher’s exact test were performed in R-2.3.2 using “cor()” and “fisher.test()” command, respectively. Student’s *t* test was performed in GraphPad Prism 5.0 (GraphPad Software, La Jolla, CA, USA).

## Results

### Bulk-cell target deep DNA sequencing revealed no evidence for BCSC phenotype-associated genetic variants

**Fig. 1:**
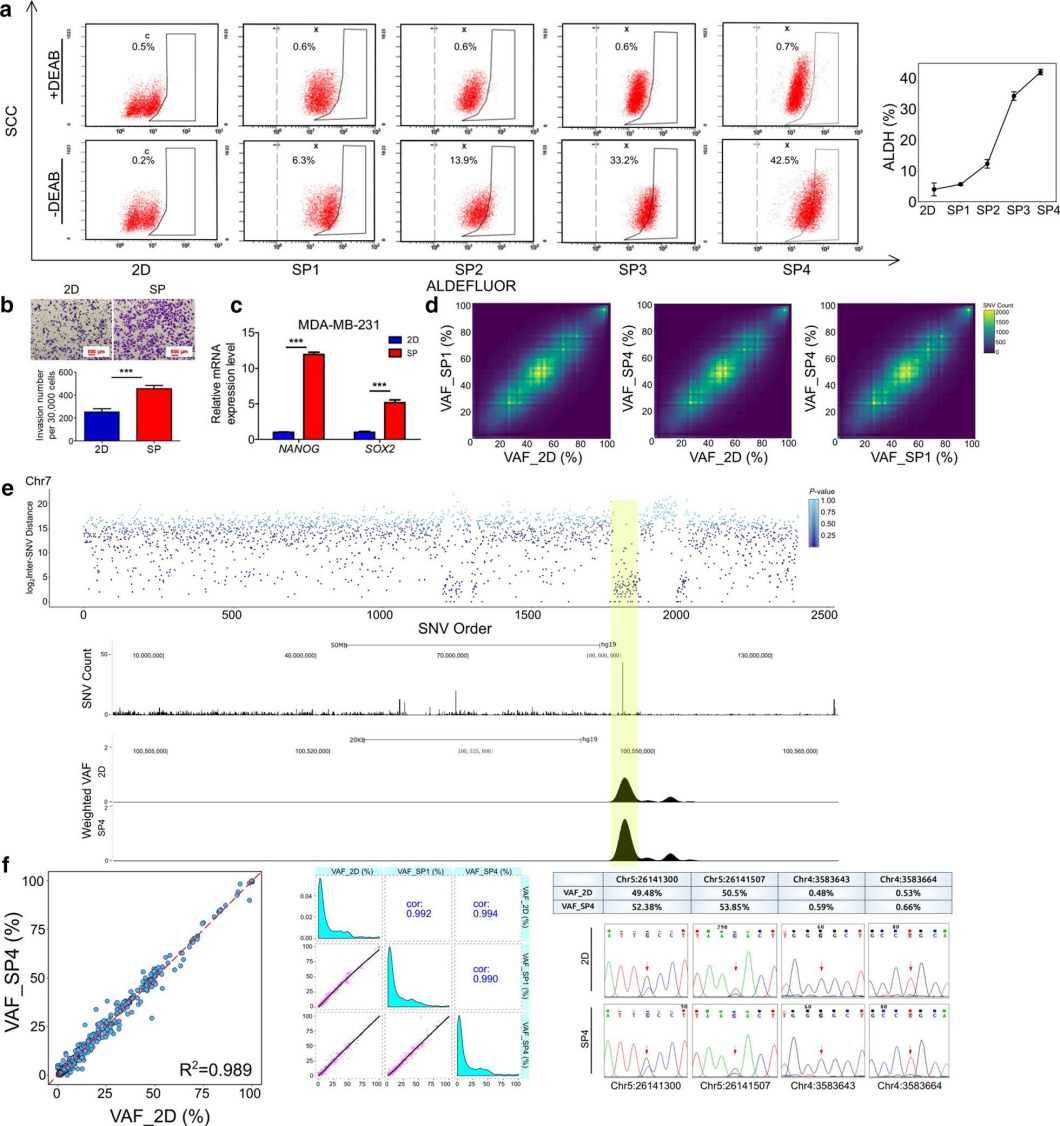
Identification and investigation of potential breast cancer stem cell (BCSC)-associated mutation hotspots. **a** Ascending trend of the percentage of the aldehyde dehydrogenase (ALDH)-positive cell population across the samples from the breast cancer cell line MDA-MB-231. **b** The invasion ability of enriched spheres was analyzed by transwell invasion assay. ***P < 0.001, two-tailed Student’s *t* tests. Error bars represent mean ± standard deviation (SD). **c** Expression levels of markers related to cancer stem cells [nanog homeobox (*NANOG*) and SRY (sex determining region Y)-box 2 (*SOX2*)] were assessed by real-time quantitative PCR in both enriched spheres (SP) and monolayer parental cells (2D). ***P < 0.001, two-tailed Student’s *t*-tests. Error bars represent mean ± SD. **d** Histogram 2D plots, conducted by the R package “plotly”, show the comparison of variant allele frequency (VAF) between every two samples. The VAF of most single nucleotide variant (SNV) sites in the whole genome is observed as being similar. **e** One hotspot region in chromosome 7 highlighted with a yellow bar is displayed as an example. First, potential SNV sites along the genome were ordered from the first to the last variant on chromosome 7 and colored according to *P* values. The distance between each mutation and the one prior to it (the inter-SNV distance) is plotted on the vertical axis (rainfall plot). *P* values were determined by an exponential distribution formula. Additionally, the number of potential SNV sites of each bin was visualized by University of California Santa Cruz Genome Browser (GB), with the whole chromosome divided into 10,000 equal bins. Next, hotspots of parental cells (2D), and derived spheres of the fourth generation (SP4) hotspot was displayed by GB using the sliding window approach, which was performed by shifting one base each time along the chromosome from start to end and calculating the SNV density and VAF level in each 1000 bp window. **f** Target deep DNA sequencing of comparison of VAF between every two samples revealed no difference from 2D to SP4 (left and middle). R^2^ was determined by regression analysis. Cor denotes the Pearson correlation coefficient. The dotted line represents the diagonal line. Sanger sequencing validated part of the results of target deep DNA sequencing (right)

Serial sphere formation assay was performed to enrich BCSCs of the breast cancer cell line MDA-MB-231. Then, an ALDEFLUOR assay was performed to investigate the proportion of BCSCs. Compared with the parental cells grown in a monolayer culture, spheres displayed gradually increased percentage of ALDH-positive cells, with almost half of the spheres of the fourth generation being composed of BCSCs (Fig. 1a; Additional file 7: Figs. S2a, S2b). In addition, compared with parental cells, spheres exhibited an obviously increased invasive capacity and higher expression of cancer stem cell markers (Fig. 1b, 1c, Additional file 7: Fig. S2c). Then, we collected the parental cells (2D) and derived spheres of the first generation (SP1) and fourth generation (SP4) for the WGS analysis. We assumed that if BCSCs were to be associated with particular genetic alterations, then the proportion of SNVs which BCSC population specifically carried would increase from 2D to SP4, leading to an increased VAF of these SNVs. Therefore, the SNVs with increased VAF from 2D to SP4 should be the genetic basis of BCSCs. However, the VAF of most SNV sites in the whole genome were similar in both 2D and SP4 (Fig. 1d).

To determine the SNV sites with significantly increased VAF, we performed Fisher’s exact test on the read counts supporting the variant in each SNV site between 2D and SP4. To find out whether these SNVs were evenly distributed or spatially clustered, we developed a hotspot-calling algorithm based on the fact that the distances between every two adjacent potential SNV sites follow exponential distribution (Additional file 7: Fig. S1a). Hotspots were defined as the regions with SNV sites more densely distributed than statistically expected (Fig. 1e; Additional file 7: Fig. S1b), representing the most likely regions harboring genetic variants associated with BCSCs.

To determine whether genomic alteration contributed to the BCSC phenotype and to investigate the genetic basis associated with BCSCs, we performed target deep DNA sequencing in bulk cells on 2D, SP1, and SP4. Target deep DNA sequencing covering all the hotspots yielded a median of 4500-fold coverage per site (Additional file 7: Fig. S3); however, no difference was found in the VAF from 2D to SP4 (Fig. 1f), making the hypothesis of heritable genetic changes contributing to the BCSC phenotype unlikely.

### Single-cell target deep DNA sequencing confirmed the absence of significant genetic difference between BCSCs and non-BCSCs

To understand BCSC at a single-cell level, we turned to single-cell sequencing on non-BCSCs and BCSCs, with non-BCSC denoted as a single cell that cannot give rise to spheres and BCSC denoted as the sphere derived from a single cell (Additional file 7: Fig. S4). Single-cell target deep DNA sequencing was performed on both the hotspots we identified in the WHM panel (Additional file 5: Table S5) and the CHM panel (Additional file 2: Table S2).

**Fig. 2:**
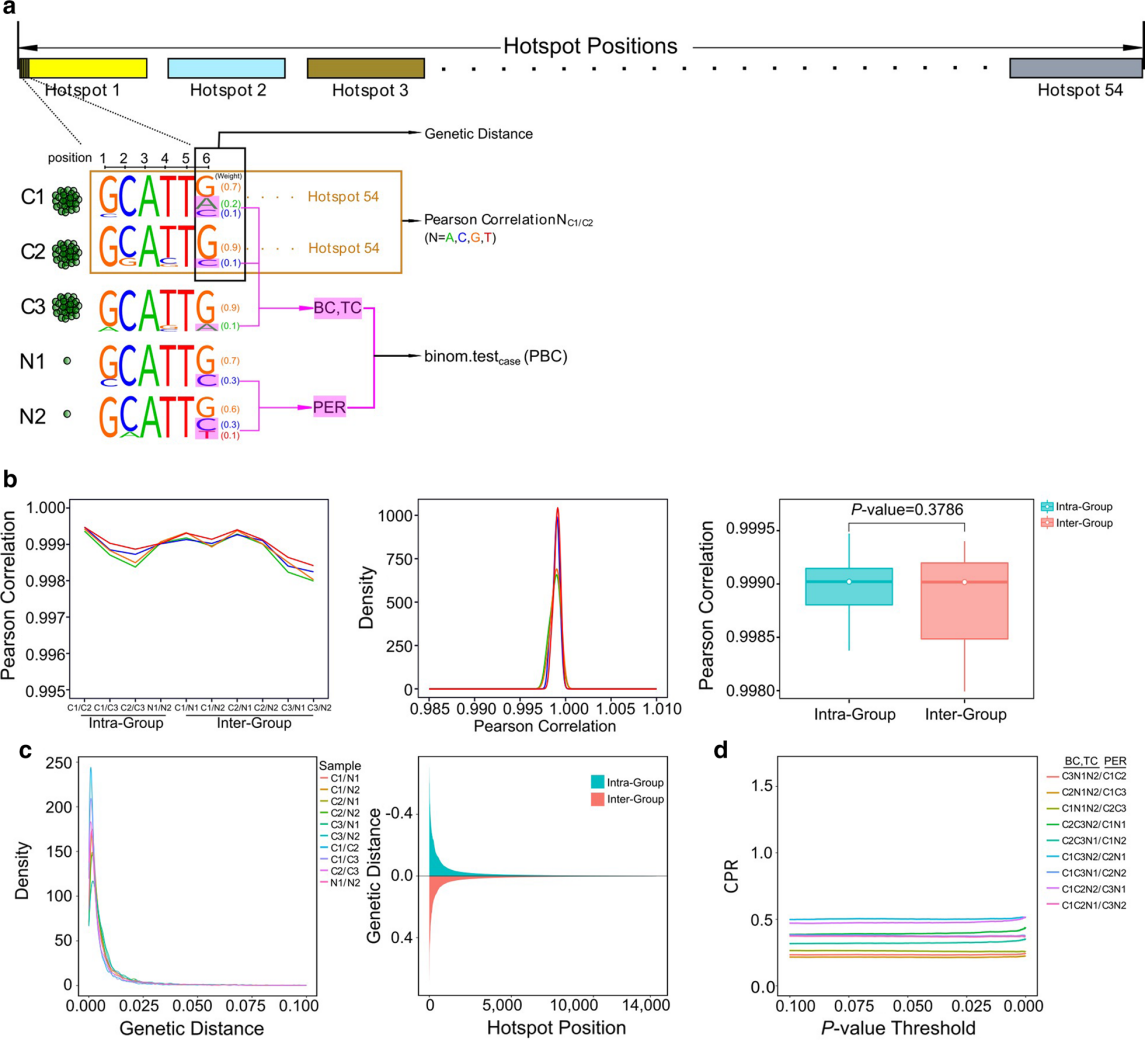
Single-cell target deep DNA sequencing of BCSCs and non-BCSCs. **a** Schematic depiction of single-cell target deep DNA sequencing analysis. Pearson correlations between every two samples were determined by the base weight, i.e., the fraction of a base in all four possible bases, at each position in hotspot regions. Binomial test was used to assess the probability of background count (PBC) in the 3 BCSCs from a binomial distribution with the position error rate (PER) determined by 2 non-BCSCs. A PBC lower than the threshold (0.01 here) denotes that the alternative reads cannot all be generated by sequencing errors, i.e., a true SNV is called. **b** Extremely high Pearson correlations of the genomic program between every two samples (left and middle). The box plot shows no significant differences between the correlation of inter-group samples and that of intra-group samples (right). The *P* value was determined by a two-tailed *t* test. **c** The distribution of genetic distances of each site between every two samples is in a narrow range (left), showing no difference between the inter-group and intra-group at all hotspot sites (ordered by the genetic distance, right). **d** Constant trend of case-permutation ratio (CPR) of each group following adjustment of the *P* value threshold. CPR was defined as the ratio of the number of sites with *P* values less than a threshold in the case group to permutation group

The landscape and general approach for the single-cell DNA sequencing analysis was exemplified by the WHM panel (Fig. 2a). Target deep DNA sequencing of the WHM panel in 5 samples yielded a median of 4000-fold coverage per site (Additional file 7: Fig. S5). On the basis of the extremely high correlation coefficient of the base weight between every two samples and the lack of a significant difference (*P* = 0.379) between the inter-group (BCSC versus non-BCSC) and intra-group (BCSC versus BCSC or non-BCSC versus non-BCSC) (Fig. 2b; Additional file 7: Fig. S6), we inferred the identical genetic spectrum across the 5 samples. On the other hand, all the genetic distances, a metric to measure nucleotide composition pattern differences between two samples, were extremely small with an average of approximately 0.001 for both inter-group and intra-group samples (Fig. 2c), further indicating the reliability of the result.

To systematically compare the genomic program between BCSCs and non-BCSCs and distinguish the genuine variants from sequencing artifacts, we developed a method based on quantification of the error rate for each base position. This approach assessed PBC in the 3 BCSCs from a binomial distribution with PER determined by 2 non-BCSCs. It indicated whether it was possible to observe the amount of variant alleles at a genomic position where no genuine variant exist. Thereafter, 764 positions of all 13,855 WHM sites were flagged as potential mutation sites with a *P* value less than 7.218 × 10^−8^ (0.001/13,855). To quantify the amount of false positives, a permutation analysis was performed on the 5 samples. For each permutation, we randomly chose 2 samples to calculate PER, and the rest 3 samples were used to calculate PBC. The numbers of potential mutation sites of each permutation were similar. Furthermore, with the threshold *P* value varying from 0 to 0.1, the numbers of mutation sites in both permutation group and case group were evenly reduced, leading to a consistent and constant trend of CPR (Fig. 2d). Therefore, we concluded that the dissimilarity between the genome of BCSCs and non-BCSCs was due to technical noise, predicting no evidence for genomic changes in BCSCs. The conclusion was also supported by the target deep DNA sequencing of the CHM panel (Additional file 7: Figs. S7–9).

### scRNA-seq showed that self-renewal capability was marked by a distinct profile of gene expression

**Fig. 3:**
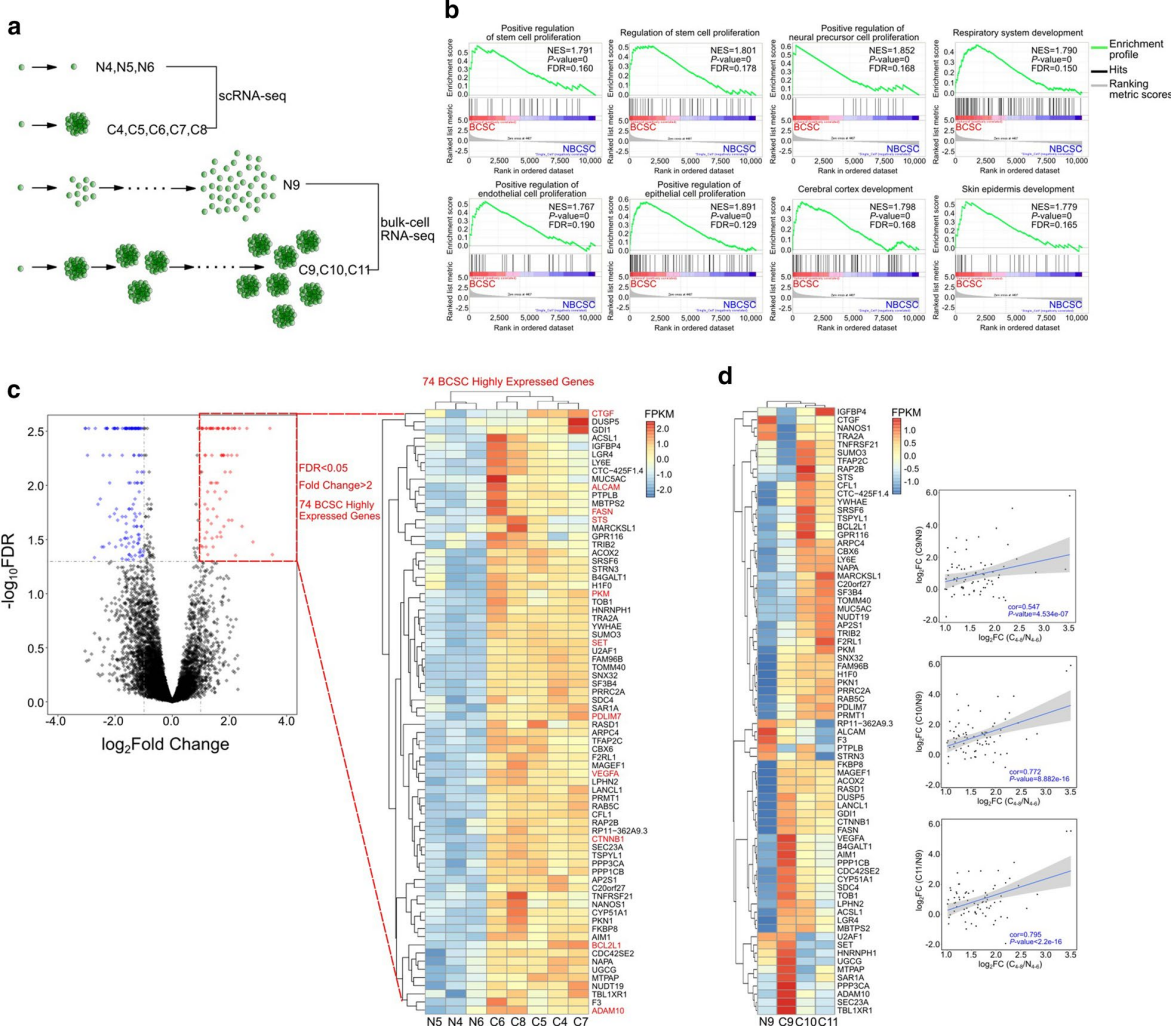
Single-cell RNA sequencing (scRNA-seq) and gene differential expression analysis. **a** Schematic depiction of the origination of sequenced samples. **b** Gene set enrichment analysis (GSEA) of gene sets enriched in BCSCs compared with those in non-BCSCs. FDR, false discovery rate; NES, normalized enrichment score. **c** The dot plot (left) shows differentially expressed genes between BCSCs and non-BCSCs. The red dots represent 74 BCSC highly expressed genes with a false discovery rate (FDR) < 0.05 and a fold change > 2. Heatmap (right) illustrates the hierarchical clustering of BCSCs and non-BCSCs showing the 74 genes, with previously reported BCSC-associated genes highlighted with red color. **d** The validation result of BCSC highly expressed genes using bulk-cell RNA-seq. Heatmap (left) shows the relative expression of BCSC highly expressed genes, and scatter plots (right) illustrate the high correlation of the results between scRNA-seq and bulk-cell RNA-seq

We then wondered whether BCSCs possess characteristic differences in single-cell gene expression. Multiple single cells (approximately 1000) isolated from the MDA-MB-231 cell line were cultured in a sphere condition of 1 cell/well, with a very small proportion of the single cells giving rise to spheres (Fig. 3a), indicating that sporadic cells had a BCSC property characterized by a distinct profile of gene expression. To identify the transcriptional program associated with BCSCs, 5 single cell-derived spheres (BCSCs) and 3 single cells that could not give rise to sphere (non-BCSCs) were subjected to scRNA-seq. Notably, as revealed by GSEA associated with biological processes (MSigDB: Gene Ontology Biological Process), the “regulation of stem cell proliferation” and other sets were strongly enriched for the group of spheres (Fig. 3b), illustrating that the difference in single-cell gene expression was associated with BCSCs.

Next, 74 genes with significantly higher expression in BCSCs (BCSC highly expressed genes) than in non-BCSCs were identified (Fig. 3c). Notably, most of the 74 genes showed similar expression pattern as in multiple bulk-cell RNA-seq results, and the correlation of scRNA-seq and bulk-cell RNA seq results was high, illustrating the reliability of the 74 genes identified by scRNA-seq (Fig. 3a, d). Among them, we recovered well-known markers and related genes of cancer stem cells, including activated leukocyte cell adhesion molecule (*ALCAM*) [20–40], pyruvate kinase (*PKM*) [41], fatty acid synthase (*FASN*) [42], vascular endothelial growth factor (*VEGFA*) [43–45], a disintegrin and metalloproteinase domain-containing protein 10 (*ADAM10*) [46], B cell lymphoma 2 like 1 (*BCL2L1*) [47–50], connective tissue growth factor (*CTGF*) [51], catenin beta 1 (*CTNNB1*) [52, 53], PDZ and LIM domain protein 7 (*PDLIM7*) [54–59], steroid sulfatase (*STS*) [60–64], and SET nuclear proto-oncogene (*SET*) [65–67].

### Exploring BCSC highly expressed genes for BCSC markers

**Fig. 4:**
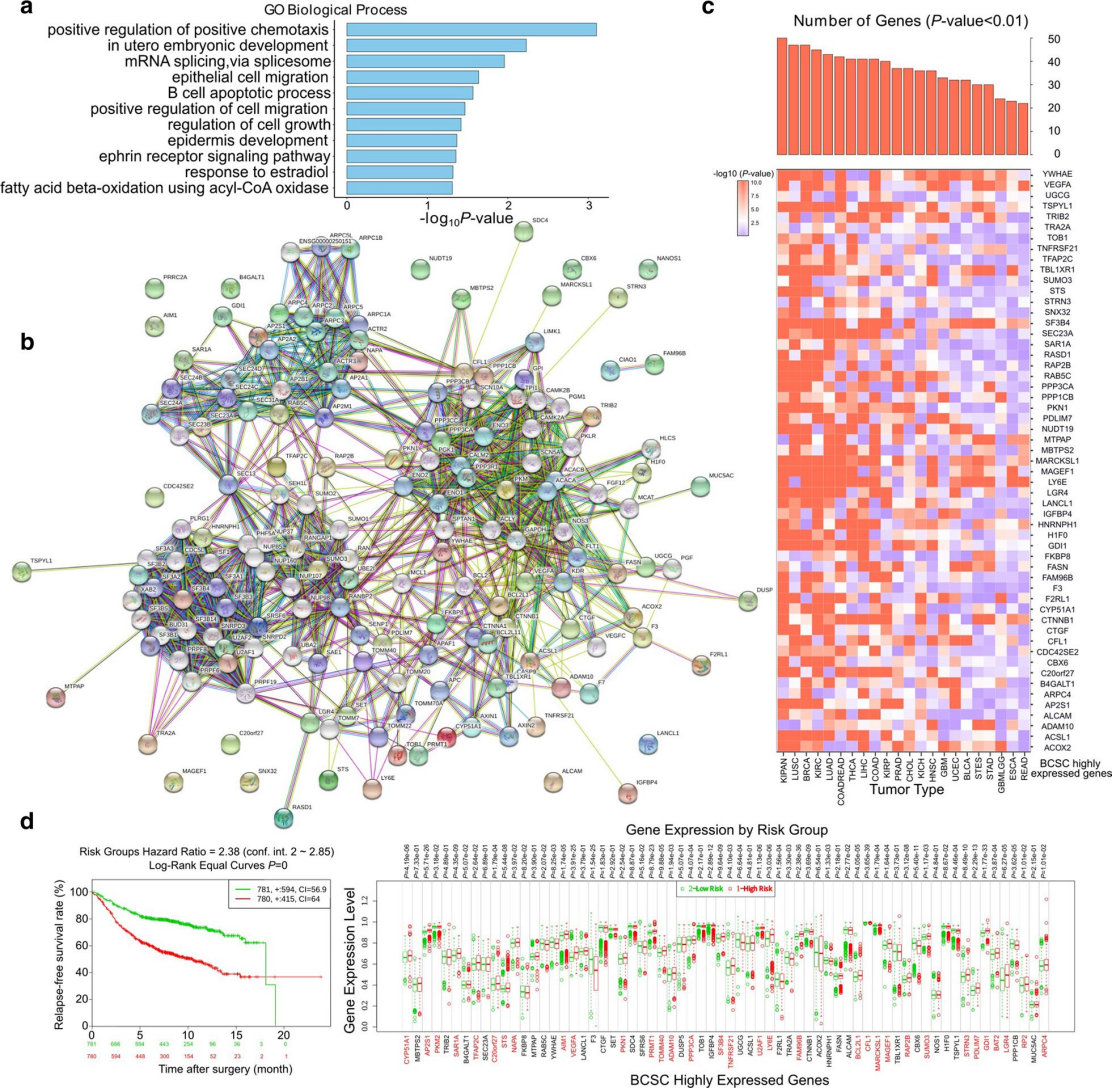
Biological and clinical significance of the BCSC highly expressed genes. **a** Gene Ontology (GO) analysis of the BCSC highly expressed genes in biological process. *P* values (one-tail Fisher exact *P* values used for gene enrichment analysis) were calculated in the DAVID database (https://david.ncifcrf.gov/tools.jsp). **b** Interaction network of BCSC highly expressed genes integrated from the STRING database. Network nodes represent genes, and edges represent gene–gene associations. A detailed legend is available at https://string-db.org. **c** Investigation of the clinical relevance of BCSC highly expressed genes in 22 cancer types. The expression of each gene in cancer and corresponding normal tissues was analyzed by a two-tailed *t* test. Heatmap was horizontally sorted by the number of genes with *P* < 0.01 in a particular cancer type, shown as red columns on the top. **d** Kaplan–Meier relapse-free survival curve (left) of patients with low (green) and high (red) risk grouped by BCSC highly expressed genes in SurvExpress (dataset: Breast cancer relapse data). The total number of each group was shown in the top right corner, and the number of censoring samples are marked with a “+” symbol. The concordance index (CI) per curve was also included. The *P* value was determined by a log-rank test. The x axis represents the years of the study. In rows and corresponding colors, the numbers of samples not presenting the event at the matching time are shown. The box plot (right) shows the comparison of the gene expression between the low- and high-risk groups. Genes significantly (*P* < 0.05) highly expressed in the high-risk group are highlighted in red. *P* values were calculated using two-tailed *t* test

Functionally, Gene Ontology (GO) analysis indicated that the 74 BCSC highly expressed genes were associated with embryonic development, epithelial cell migration, and positive regulation of cell migration, as expected from BCSCs (Fig. 4a). Additionally, the functional networks involved in the 74 genes were determined (protein–protein interaction enrichment *P* value = 0.004) in STRING datasets, indicating that the genes were biologically connected and coordinated and revealing the core functional networks underlying positive regulation of the cellular process (FDR = 0.003) and cell surface receptor signaling pathway (FDR = 0.003) (Fig. 4b), suggesting the possibility of these genes being BCSC markers. Furthermore, to evaluate whether BCSC highly expressed genes generated from the in vitro model had clinical relevance, we queried TCGA datasets across multiple cancer types. Most of the genes manifested significant differential expression between cancer and normal tissues, further suggesting the feasibility of them being BCSC markers (Fig. 4c; Additional file 7: Fig. S10a).

To systematically assess the prognostic significance of the BCSC markers we identified, we performed survival analysis of breast cancer patients using the SurvExpress database. A significant prognostic association was observed, and most of the genes were expressed at higher levels in the high-risk relapse group than in the low-risk relapse group, indicating their value in predicting adverse prognosis (Fig. 4d). Additionally, individual inspection of these genes further confirmed their prognostic significance in breast cancer (Additional file 7: Fig. S11). Moreover, the clustering of PRECOG *z* scores [17] revealed prognostic specificity across distinct cancer types and subtypes, with neuroblastoma being the most affected (Additional file 7: Fig. S10b). Taken together, our results highlight the clinical significance of these BCSC markers, suggesting novel targets for anticancer therapy.

## Discussion

Cancer stem cells can result from cancer cells by acquiring mutations [68, 69], but in other cases there is no clear genetic cause, raising the possibility of non-genetic cell transcriptomic variability [70–72]. The present work aimed at a better understanding of cancer stem cells. Breast cancer, which often relapses and metastasizes, is a paradigmatic example for studying cancer stem cells. Serial sphere formation assay, a widely used in vitro technique for assessing self-renewal capacity [73, 74], was performed to enrich BCSCs. Recent works have demonstrated that there were no significant tumorigenicity differences between ALDH^+^CD44^+^CD24^−^ cell population and ALDH^+^ CD44^−^CD24^−^ cell population [75] and that ALDH^−^ cells bearing the CD44^+^CD24^−^/lin^−^ phenotype was not tumorigenic. By contrast, the ALDH^+^ population that did not display the CD44^+^/CD24^−^/lin^−^ phenotype was capable of generating tumors [76]. Taken together, it is suggestive that high ALDH activity can be considered as an identifier of the tumorigenic cell fraction, capable of self-renewal and generating tumors. In the present study, we used ALDH in FACS assay to investigate the proportion of BCSCs in spheres. To investigate the genetic basis of BCSCs origination, we determined the potential mutation hotspot regions using WGS, and performed target deep DNA sequencing on parental cancer cells and derived serial-generation spheres in increasing order of BCSC frequency, which revealed no validated genetic changes specifically associated with BCSCs. With respect to the different VAF provided by WGS and target deep DNA sequencing, we suggest that the latter quantifies VAF more precisely by generating deeper genome coverage. Previous studies also applied target deep DNA sequencing to examine clonal evolution through its unequivocal evidence of defining VAF [77, 78].

Population-based sequencing indicated no clear genetic cause contributing to BCSC. To understand BCSCs at the single-cell level, single-cell DNA sequencing was performed on non-BCSCs and BCSCs. All cells of one sphere were derived from the originally seeded single cell, leading to a homogenous genome within one sphere. Thus, sequencing of the sphere increased the accuracy in identifying variants without the disturbances of genomic heterogeneity. Nevertheless, single-cell DNA sequencing also have some limitations, for instance, a fraction of stochastic allele-alterations could be introduced by the process of whole genome amplification [79]. Concerned with this issue, we performed single-cell target deep DNA sequencing for the WHM and CHM panels. The generation of high-depth sequencing data allowed us to accurately quantify the allele frequency in all samples, permitting the calculation of a base weight for each site in a hotspot region. On the basis of base weight of each site, we obtained the correlation coefficients and genetic distances to globally assess the genomic program across all samples. Moreover, a method based on quantification of error rate of each base position was developed, allowing us to systematically analyze the genomic program. Taken together, single-cell target deep DNA sequencing confirmed the absence of significant genetic difference between BCSCs and non-BCSCs.

The emergence of self-renewal capability is a complex process involving transcriptomic variability. Profiling the transcriptomes of individual cells via scRNA-seq allowed the functional role of heterogeneity in gene expression levels between cells to be investigated [80]. Bioinformatics analysis identified 74 candidate BCSC highly expressed genes through single-cell transcriptome sequencing. These 74 genes overlapped with some genes identified by other studies [41–47], and many extend beyond breast cancer, suggesting the existence of a general expression program co-opted in the cancer stem cell phenotype. Overall, the present study explored the identity of BCSC and provided a framework for understanding BCSC.

Here, we documented proof of the concept that a non-genetic cause leads to BCSCs, suggestive of common, shared epigenetic regulation that contributes to the BCSC phenotype. Further elucidation of the reprogramming and plasticity of switching between BCSC and non-BCSC states at an epigenetic level may open new avenues for therapeutic targeting. For instance, epigenetic activation of twist family bHLH transcription factor 1 (TWIST1) by metadherin (MTDH) promotes cancer stem-like cell traits in breast cancer [12]. MTDH activates TWIST1 expression indirectly by facilitating histone H3 acetylation on the TWIST1 promoter, a process mediated by the histone acetyltransferase cAMP-response element binding protein (CREB)-binding protein (CBP). Similarly, poised chromatin at the zinc finger E-box binding homeobox 1 (ZEB1) promoter enables breast cancer cell plasticity and enhances tumorigenicity [13], supporting a dynamic model in which interconversions between low and high tumorigenic states occur frequently, thereby increasing tumorigenic and malignant potential. Therefore, the roles of the epigenetics are being increasingly required for phenotypic plasticity, specifically in a context where genome sequences are not altered. In addition, epigenetic and genetic causes of BCSC are not mutually exclusive. Epigenetic effects may provide the initial BCSC state, allowing a small subpopulation of tumor cells to potentially self-renew until some acquire secondary mutations that drive cancer progression to relapse. The unification of in vivo studies will allow for a more comprehensive description of BCSCs.

## Conclusions

The present study demonstrated that no genetic changes contributed to the BCSC identity. Breast cancer cells displayed transcriptomic variability at the single-cell level and determined BCSC phenotype. The single-cell transcriptomic variability involved coordinated transcription of a number of BCSC markers and was found to be significantly associated with clinical prognosis.

## Additional files

**Additional file 1: Table S1.** Primer sequences for real-time quantitative PCR.

**Additional file 2: Table S2.** Amplicon primers designed for the WGS hotspot mutation (WHM) panel.

**Additional file 3: Table S3.** Targeted genes and mutations in the cancer hotspot mutation (CHM) panel.

**Additional file 4: Table S4.** Amplicon primers designed for the CHM panel.

**Additional file 5: Table S5.** The WHM panel of hotspots identified by whole-genome sequencing (WGS).

**Additional file 6: Table S6.** Definition of case groups and permutation groups.

**Additional file 7: Fig. S1.** Potential mutation hotspots associated with breast cancer stem cells (BCSCs) are identified by bulk-cell whole-genome sequencing (WGS). **a,** Distances of potential single nucleotide variations (SNV) sites follow an exponential distribution. **b,** Two hotspots in chromosome 6 highlighted with a yellow bar are displayed as an example. **c and d,** Distribution of length (C) and proportion of functional annotations (D) for hotspots. **Fig. S2.** This figure related to Figure 1A. Serial sphere formation assay. **a,** Serial sphere formation assay from the first to fourth generation was performed in MDA-MB-231 cells. The spheres were photographed using an inverted microscope (Olympus). Scale bar, 200 μm. **b,** Cell number of spheres from the first to fourth generation. **c**, Expression levels of markers related to cancer stem cells [nanog homeobox (NANOG) and SRY (sex determining region Y)-box 2(SOX2)] was assessed by western blot assay in both enriched spheres (SP) and monolayer parental cells (2D). **Fig. S3.** Bulk-cell target deep DNA sequencing data evaluation. The violin plot (A) illustrates the distribution of depth in the target deep DNA sequencing, and the reads coverage distribution of each hotspot are shown by the pile-up bar plots (B). **Fig. S4.** Single-cell sphere formation assay. Images of single cell-derived spheres (red, BCSCs) and single cells that could not form spheres (green, non-BCSCs). The spheres and single cells were photographed using an inverted microscope (Olympus). Scale bar, 50 μm. **Fig. S5.** Data evaluation of single-cell target deep DNA sequencing of the hotspot region panel. **a and b,** Depth distribution of target deep DNA sequencing of hotspots from 5 samples. **c and d,** Reads coverage distribution of hotspots. **Fig. S6.** Pearson correlations of the genomic program (the hotspot region panel) between every two samples. **Fig. S7.** Data evaluation of single-cell target deep DNA sequencing of the cancer hotspot mutation (CHM) panel. **a and b,** Depth distribution of target deep DNA sequencing of hotspots from 5 samples. **c,** Reads coverage distribution of hotspots. **Fig. S8.** Pearson correlations of the genomic program (the CHM panel) between every two samples. **Fig. S9.** Single-cell target deep DNA sequencing of the CHM panel confirms no significant difference between BCSCs and NBCSCs. **Fig. S10.** Clinical significance of the BCSC highly expressed genes in pan-cancer. **a,** The expression of each gene in cancer and corresponding normal tissues was analyzed by a two-tailed Student’s *t* test. The heatmap is vertically sorted by the number of cancer types with fold change (FC) < -2 or FC > 2 shown as red columns in the right. **b,** Hierarchical clustering of PRECOG z scores is shown by heatmap. **Fig. S11.** Prognosis significance of the BCSC highly expressed genes in breast cancer. Kaplan-Meier curves of estimated relapse-free survival (RFS) for breast cancer patients with low (black) and high (red) expression of BCSC highly expressed genes in the Kaplan-Meier database. HR, hazard ratio. *P* values were determined by log-rank test.

## Abbreviations

BCSCs: breast cancer stem cells
NGS: next-generation sequencing
WGS: whole-genome sequencing
DEAB: diethylaminobenzaldehyde
RLE: run-length encoding
MDA: multiple displacement amplification
WGA: whole genome amplification
TC: total count
MC: major count
BC: background count
PER: position error rate
PBC: probability of background count
CPR: case-permutation ratio
NP: number of positions
DEG: differentially expressed gene
FC: fold change
FDR: false discovery rate
SNVs: single nucleotide variations
VAF: variant allele frequency
GSEA: gene set enrichment analysis
GO: gene Ontology
GB: genome Browser
NES: normalized enrichment score
CI: concordance Index

## References

[1] Geng SQ, Alexandrou AT, Li JJ (2014). Breast cancer stem cells: multiple capacities in tumor metastasis. Cancer Lett.

[2] Kotiyal S, Bhattacharya S (2014). Breast cancer stem cells, emt and therapeutic targets. Biochem Biophys Res Commun.

[3] Liu S, Wicha MS (2010). Targeting breast cancer stem cells. J Clin Oncol.

[4] Cicalese A, Bonizzi G, Pasi CE (2009). The tumor suppressor p53 regulates polarity of self-renewing divisions in mammary stem cells. Cell.

[5] Spike BT, Engle DD, Lin JC (2012). A mammary stem cell population identified and characterized in late embryogenesis reveals similarities to human breast cancer. Cell Stem Cell.

[6] Brooks MD, Burness ML, Wicha MS (2015). Therapeutic implications of cellular heterogeneity and plasticity in breast cancer. Cell Stem Cell.

[7] Sansone P, Ceccarelli C, Berishaj M (2016). Self-renewal of cd133(hi) cells by il6/notch3 signalling regulates endocrine resistance in metastatic breast cancer. Nat Commun.

[8] Boo L, Ho WY, Ali NM (2016). Mirna transcriptome profiling of spheroid-enriched cells with cancer stem cell properties in human breast mcf-7 cell line. Int J Biol Sci.

[9] Okuda H, Kobayashi A, Xia B (2012). Hyaluronan synthase has2 promotes tumor progression in bone by stimulating the interaction of breast cancer stem-like cells with macrophages and stromal cells. Cancer Res.

[10] Dave B, Migliaccio I, Gutierrez MC (2011). Loss of phosphatase and tensin homolog or phosphoinositol-3 kinase activation and response to trastuzumab or lapatinib in human epidermal growth factor receptor 2-overexpressing locally advanced breast cancers. J Clin Oncol.

[11] Foulkes WD, Stefansson IM, Chappuis PO (2003). Germline brca1 mutations and a basal epithelial phenotype in breast cancer. J Natl Cancer Inst.

[12] Liang Y, Hu J, Li J (2015). Epigenetic activation of twist1 by mtdh promotes cancer stem-like cell traits in breast cancer. Cancer Res.

[13] Chaffer CL, Marjanovic ND, Lee T (2013). Poised chromatin at the zeb1 promoter enables breast cancer cell plasticity and enhances tumorigenicity. Cell.

[14] Subramanian A, Tamayo P, Mootha VK (2005). Gene set enrichment analysis: a knowledge-based approach for interpreting genome-wide expression profiles. Proc Natl Acad Sci USA.

[15] Lanczky A, Nagy A, Bottai G (2016). Mirpower: a web-tool to validate survival-associated mirnas utilizing expression data from 2178 breast cancer patients. Breast Cancer Res Treat.

[16] Brohee S, van Helden J (2006). Evaluation of clustering algorithms for protein-protein interaction networks. BMC Bioinform.

[17] Gentles AJ, Newman AM, Liu CL (2015). The prognostic landscape of genes and infiltrating immune cells across human cancers. Nat Med.

[18] Aguirre-Gamboa R, Gomez-Rueda H, Martinez-Ledesma E (2013). Survexpress: an online biomarker validation tool and database for cancer gene expression data using survival analysis. PLoS ONE.

[19] Pecuchet N, Rozenholc Y, Zonta E (2016). Analysis of base-position error rate of next-generation sequencing to detect tumor mutations in circulating DNA. Clin Chem.

[20] Botchkina GI, Zuniga ES, Das M (2010). New-generation taxoid sb-t-1214 inhibits stem cell-related gene expression in 3d cancer spheroids induced by purified colon tumor-initiating cells. Mol Cancer.

[21] Botchkina IL, Rowehl RA, Rivadeneira DE (2009). Phenotypic subpopulations of metastatic colon cancer stem cells: genomic analysis. Cancer Genom Proteom.

[22] Giampieri R, Scartozzi M, Loretelli C (2013). Cancer stem cell gene profile as predictor of relapse in high risk stage ii and stage iii, radically resected colon cancer patients. PLoS ONE.

[23] Haraguchi N, Ishii H, Mimori K (2013). Cd49f-positive cell population efficiently enriches colon cancer-initiating cells. Int J Oncol.

[24] Hostettler L, Zlobec I, Terracciano L (2010). Abcg5-positivity in tumor buds is an indicator of poor prognosis in node-negative colorectal cancer patients. World J Gastroenterol.

[25] Hwang Wei–Lun, Yang Muh–Hwa, Tsai Ming–Long, Lan Hsin–Yi, Su Shu–Han, Chang Shih–Ching, Teng Hao–Wei, Yang Shung–Haur, Lan Yuan–Tzu, Chiou Shih–Hwa, Wang Hsei–Wei (2011). SNAIL Regulates Interleukin-8 Expression, Stem Cell–Like Activity, and Tumorigenicity of Human Colorectal Carcinoma Cells. Gastroenterology.

[26] King JB, von Furstenberg RJ, Smith BJ (2012). Cd24 can be used to isolate lgr5 + putative colonic epithelial stem cells in mice. Am J Physiol Gastrointest Liver Physiol.

[27] Leng Z, Tao K, Xia Q (2013). Kruppel-like factor 4 acts as an oncogene in colon cancer stem cell-enriched spheroid cells. PLoS ONE.

[28] Levi E, Sochacki P, Khoury N (2014). Cancer stem cells in helicobacter pylori infection and aging: implications for gastric carcinogenesis. World J Gastrointest Pathophysiol.

[29] Lin JJ, Huang CS, Yu J (2014). Malignant phyllodes tumors display mesenchymal stem cell features and aldehyde dehydrogenase/disialoganglioside identify their tumor stem cells. Breast Cancer Res.

[30] Lugli A, Iezzi G, Hostettler I (2010). Prognostic impact of the expression of putative cancer stem cell markers cd133, cd166, cd44s, epcam, and aldh1 in colorectal cancer. Br J Cancer.

[31] Margaritescu C, Pirici D, Cherciu I (2014). Cd133/cd166/ki-67 triple immunofluorescence assessment for putative cancer stem cells in colon carcinoma. J Gastrointestin Liver Dis.

[32] Moon BS, Jeong WJ, Park J (2014). Role of oncogenic k-ras in cancer stem cell activation by aberrant wnt/beta-catenin signaling. J Natl Cancer Inst.

[33] Nautiyal J, Kanwar SS, Yu Y (2011). Combination of dasatinib and curcumin eliminates chemo-resistant colon cancer cells. J Mol Signal.

[34] Oh PS, Patel VB, Sanders MA (2011). Schlafen-3 decreases cancer stem cell marker expression and autocrine/juxtacrine signaling in folfox-resistant colon cancer cells. Am J Physiol Gastrointest Liver Physiol.

[35] Piscuoglio S, Lehmann FS, Zlobec I (2012). Effect of epcam, cd44, cd133 and cd166 expression on patient survival in tumours of the ampulla of vater. J Clin Pathol.

[36] Tachezy M, Zander H, Gebauer F (2012). Activated leukocyte cell adhesion molecule (cd166)—its prognostic power for colorectal cancer patients. J Surg Res.

[37] Tachezy M, Zander H, Wolters-Eisfeld G (2014). Activated leukocyte cell adhesion molecule (cd166): an “inert” cancer stem cell marker for non-small cell lung cancer?. Stem Cells.

[38] Yan M, Yang X, Wang L (2013). Plasma membrane proteomics of tumor spheres identify cd166 as a novel marker for cancer stem-like cells in head and neck squamous cell carcinoma. Mol Cell Proteom.

[39] Yang L, Levi E, Zhu S (2013). Cancer stem cells biomarkers in gastric carcinogenesis. J Gastrointest Cancer.

[40] Zhou J, Li P, Xue X (2013). Salinomycin induces apoptosis in cisplatin-resistant colorectal cancer cells by accumulation of reactive oxygen species. Toxicol Lett.

[41] Morfouace M, Lalier L, Oliver L (2014). Control of glioma cell death and differentiation by pkm2-oct4 interaction. Cell Death Dis.

[42] Wahdan-Alaswad RS, Cochrane DR, Spoelstra NS (2014). Metformin-induced killing of triple-negative breast cancer cells is mediated by reduction in fatty acid synthase via mirna-193b. Horm Cancer.

[43] Gokmen-Polar Y, Goswami CP, Toroni RA (2014). Gene expression analysis reveals distinct pathways of resistance to bevacizumab in xenograft models of human er-positive breast cancer. J Cancer.

[44] Yamanouchi K, Ohta T, Liu Z (2014). The wilms’ tumor gene wt1—17aa/—kts splice variant increases tumorigenic activity through up-regulation of vascular endothelial growth factor in an in vivo ovarian cancer model. Transl Oncol.

[45] Zhao D, Pan C, Sun J (2015). Vegf drives cancer-initiating stem cells through vegfr-2/stat3 signaling to upregulate myc and sox2. Oncogene.

[46] Rappa G, Mercapide J, Anzanello F (2013). Biochemical and biological characterization of exosomes containing prominin-1/cd133. Mol Cancer.

[47] Aldinucci D, Poletto D, Nanni P (2002). Cd40l induces proliferation, self-renewal, rescue from apoptosis, and production of cytokines by cd40-expressing aml blasts. Exp Hematol.

[48] Faderl S, Harris D, Van Q (2003). Granulocyte-macrophage colony-stimulating factor (gm-csf) induces antiapoptotic and proapoptotic signals in acute myeloid leukemia. Blood.

[49] Moretti L, Li B, Kim KW (2010). At-101, a pan-bcl-2 inhibitor, leads to radiosensitization of non-small cell lung cancer. J Thorac Oncol.

[50] Nanta R, Kumar D, Meeker D (2013). Nvp-lde-225 (erismodegib) inhibits epithelial-mesenchymal transition and human prostate cancer stem cell growth in nod/scid il2rgamma null mice by regulating bmi-1 and microrna-128. Oncogenesis.

[51] Edwards LA, Woolard K, Son MJ (2011). Effect of brain- and tumor-derived connective tissue growth factor on glioma invasion. J Natl Cancer Inst.

[52] Bauer L, Langer R, Becker K (2012). Expression profiling of stem cell-related genes in neoadjuvant-treated gastric cancer: a notch2, gsk3b and beta-catenin gene signature predicts survival. PLoS ONE.

[53] Chan TA, Wang Z, Dang LH (2002). Targeted inactivation of ctnnb1 reveals unexpected effects of beta-catenin mutation. Proc Natl Acad Sci USA.

[54] Abdulkarim B, Sabri S, Zelenika D (2003). Antiviral agent cidofovir decreases epstein-barr virus (ebv) oncoproteins and enhances the radiosensitivity in ebv-related malignancies. Oncogene.

[55] Du C, Wen B, Li D (2006). Downregulation of epstein-barr virus-encoded latent membrane protein-1 by arsenic trioxide in nasopharyngeal carcinoma cells. Tumori.

[56] Kondo S, Wakisaka N, Muramatsu M (2011). Epstein-barr virus latent membrane protein 1 induces cancer stem/progenitor-like cells in nasopharyngeal epithelial cell lines. J Virol.

[57] Yang CF, Peng LX, Huang TJ (2014). Cancer stem-like cell characteristics induced by eb virus-encoded lmp1 contribute to radioresistance in nasopharyngeal carcinoma by suppressing the p53-mediated apoptosis pathway. Cancer Lett.

[58] Yoshizaki T, Kondo S, Wakisaka N (2013). Pathogenic role of epstein-barr virus latent membrane protein-1 in the development of nasopharyngeal carcinoma. Cancer Lett.

[59] Zhang Q, Zhang Z, Wang C (2008). Proteome analysis of the transformation potential of the epstein-barr virus-encoded latent membrane protein 1 in nasopharyngeal epithelial cells np69. Mol Cell Biochem.

[60] Fujii T, Saito D, Yoshida S (1988). the influence of sodium thiosulfate on the antitumor effect of cisplatin in human gastric cancer cell lines. Gan To Kagaku Ryoho.

[61] Kehlen A, Greither T, Wach S (2014). High coexpression of ccl2 and cx3cl1 is gender-specifically associated with good prognosis in soft tissue sarcoma patients. Int J Cancer.

[62] MacIsaac ZM, Shang H, Agrawal H (2012). Long-term in vivo tumorigenic assessment of human culture-expanded adipose stromal/stem cells. Exp Cell Res.

[63] Mohrin M, Chen D (2013). Sirtuins, tissue maintenance, and tumorigenesis. Genes Cancer.

[64] Taubert H, Wurl P, Greither T (2007). Stem cell-associated genes are extremely poor prognostic factors for soft-tissue sarcoma patients. Oncogene.

[65] Abelson S, Shamai Y, Berger L (2013). Niche-dependent gene expression profile of intratumoral heterogeneous ovarian cancer stem cell populations. PLoS ONE.

[66] Mezencev R, Wang L, McDonald JF (2012). Identification of inhibitors of ovarian cancer stem-like cells by high-throughput screening. J Ovarian Res.

[67] Perumal D, Singh S, Yoder SJ (2012). A novel five gene signature derived from stem-like side population cells predicts overall and recurrence-free survival in nsclc. PLoS ONE.

[68] Li C, Wu S, Yang Z (2017). Single-cell exome sequencing identifies mutations in kcp, loc440040, and loc440563 as drivers in renal cell carcinoma stem cells. Cell Res.

[69] Yang Z, Li C, Fan Z (2017). Single-cell sequencing reveals variants in arid1a, gprc5a and mll2 driving self-renewal of human bladder cancer stem cells. Eur Urol.

[70] Wang Y, Cardenas H, Fang F (2014). Epigenetic targeting of ovarian cancer stem cells. Cancer Res.

[71] Shah M, Allegrucci C (2013). Stem cell plasticity in development and cancer: epigenetic origin of cancer stem cells. Subcell Biochem.

[72] Bapat SA (2013). Epigenetic regulation of cancer stem cell gene expression. Subcell Biochem.

[73] Beck B, Blanpain C (2013). Unravelling cancer stem cell potential. Nat Rev Cancer.

[74] Pastrana E, Silva-Vargas V, Doetsch F (2011). Eyes wide open: a critical review of sphere-formation as an assay for stem cells. Cell Stem Cell.

[75] Liu M, Liu Y, Deng L (2018). Transcriptional profiles of different states of cancer stem cells in triple-negative breast cancer. Mol Cancer.

[76] Ginestier C, Hur MH, Charafe-Jauffret E (2007). Aldh1 is a marker of normal and malignant human mammary stem cells and a predictor of poor clinical outcome. Cell Stem Cell.

[77] Ding L, Ley TJ, Larson DE (2012). Clonal evolution in relapsed acute myeloid leukaemia revealed by whole-genome sequencing. Nature.

[78] Ley TJ, Mardis ER, Ding L (2008). DNA sequencing of a cytogenetically normal acute myeloid leukaemia genome. Nature.

[79] Borgstrom E, Paterlini M, Mold JE (2017). Comparison of whole genome amplification techniques for human single cell exome sequencing. PLoS ONE.

[80] Kim JK, Kolodziejczyk AA, Ilicic T (2015). Characterizing noise structure in single-cell rna-seq distinguishes genuine from technical stochastic allelic expression. Nat Commun.

