# Supplementary material for: Transcriptomic but not genomic variability confers phenotype of breast cancer stem cells
Source: Cancer Commun (Lond). 2018 Sep 19;38:56. doi: 10.1186/s40880-018-0326-8 (PMC6146522; doi:10.1186/s40880-018-0326-8)
Supplement: Supplementary file 1 — Additional file 1: Table S1. Primer sequences for real-time quantitative PCR. [file 40880_2018_326_MOESM1_ESM.docx]

Table S1. Primer sequences for real-time quantitative PCR.

| **Gene** | **Primer sequences (5' to 3')** | |
| --- | --- | --- |
|  | **Forward** | **Reverse** |
| **NANOG** | **CCAAATTCTCCTGCCAGTGAC** | **CACACGTCTTCAGGTTGCAT** |
| **OCT4** | **GAGAACCGAGTGAGAGGCAACC** | **CATAGTCGCTGCTTGATCGCTTG** |
| **SOX2** | **ACTCCATGACCAGCTCGCAGA** | **GGACTTGACCACCGAACCC** |
| **ACTB** | **TTGCCGACAGGATGCAGAAGGA** | **AGGTGGACAGCGAGGCCAGGAT** |

NANOG: Nanog homeobox. OCT4: organic cation/carnitine transporter 4. SOX2: SRY (sex determining region Y)-box 2. ACTB: actin beta.
