# Supplementary figures and images for: Transcriptomic but not genomic variability confers phenotype of breast cancer stem cells
Source: Cancer Commun (Lond). 2018 Sep 19;38:56. doi: 10.1186/s40880-018-0326-8 (PMC6146522; doi:10.1186/s40880-018-0326-8)

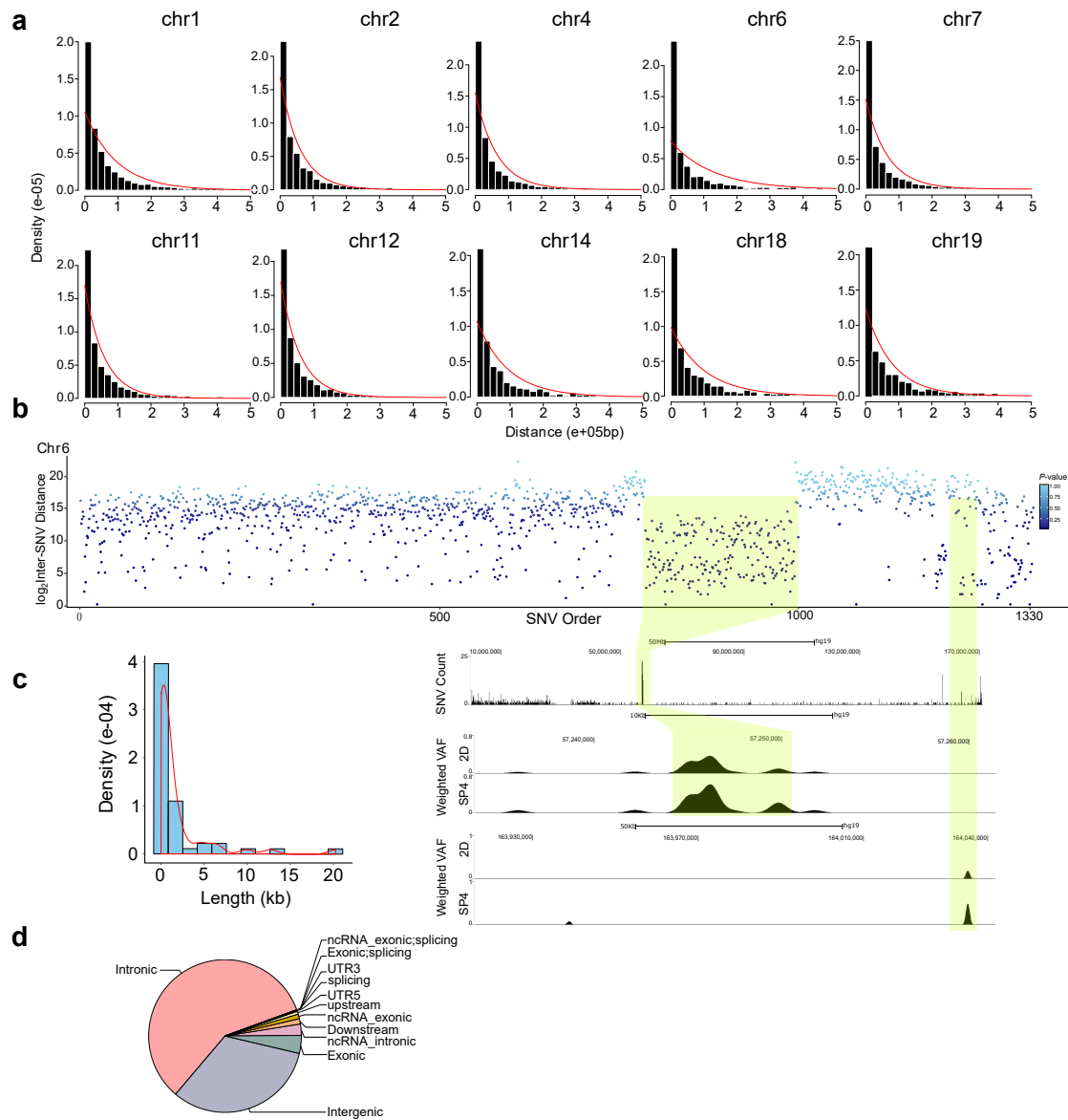

Fig. S1

**a**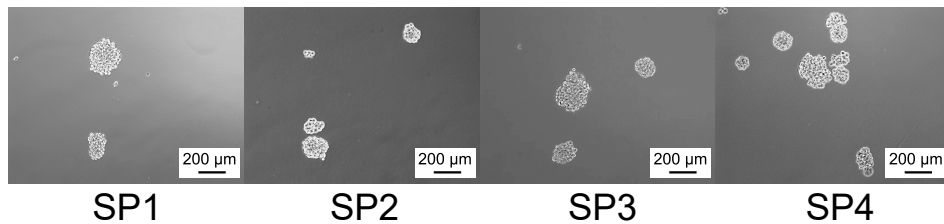**b**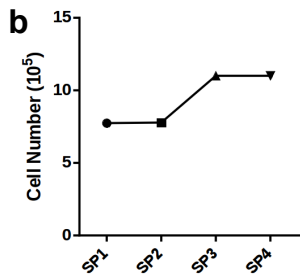**c**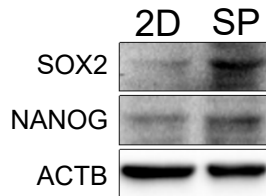

Fig. S2

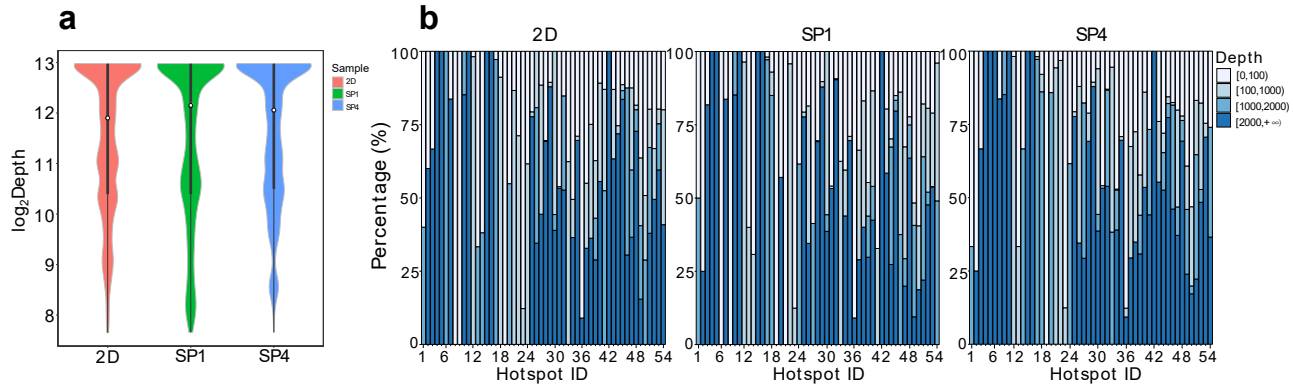

Fig. S3

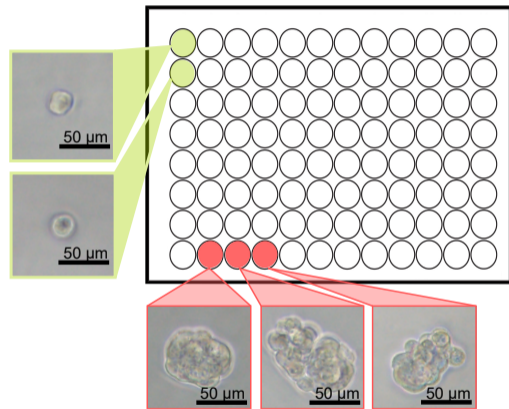

Fig. S4

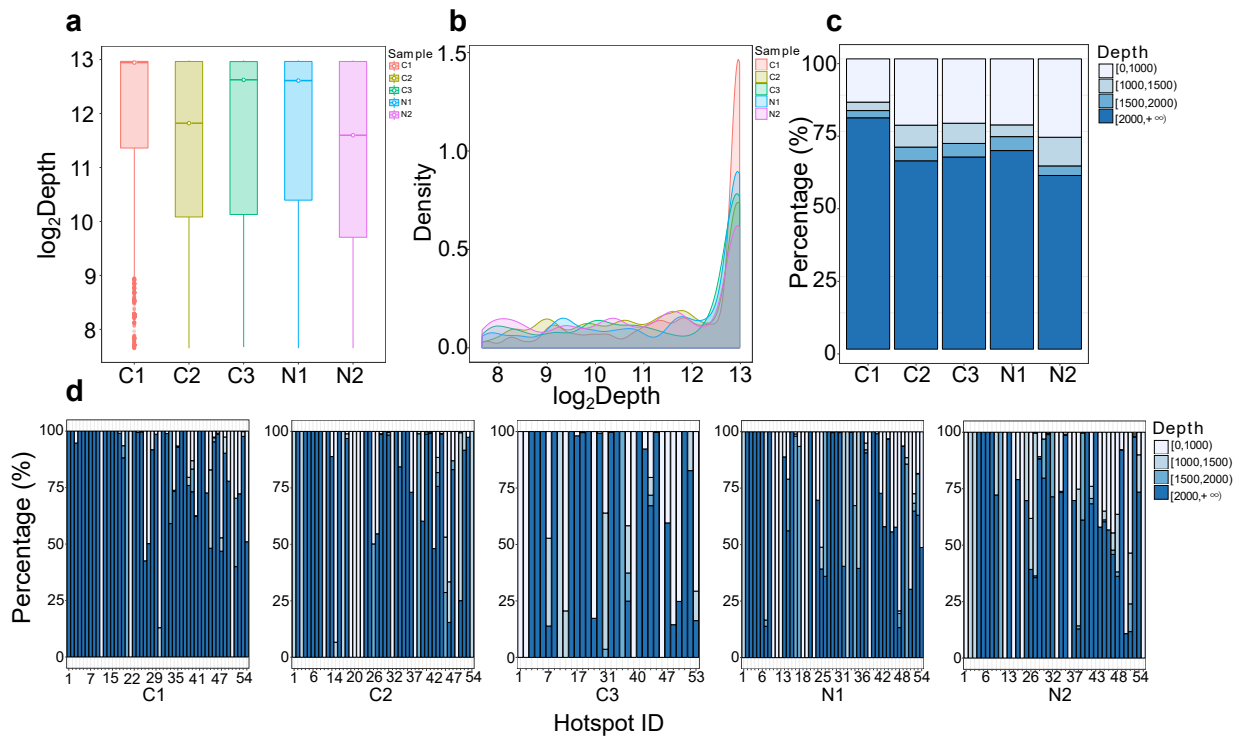

Fig. S5

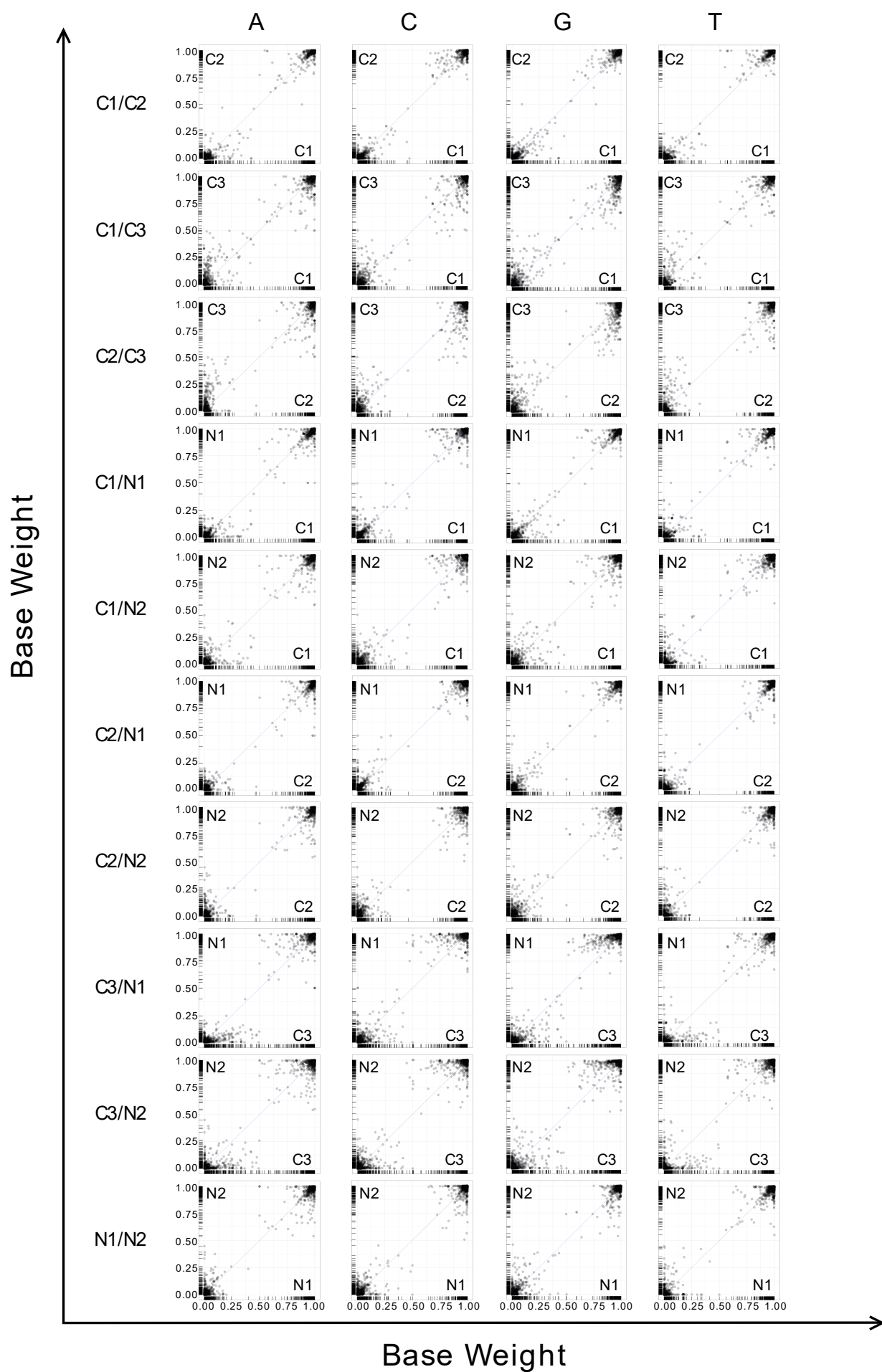

Fig. S6

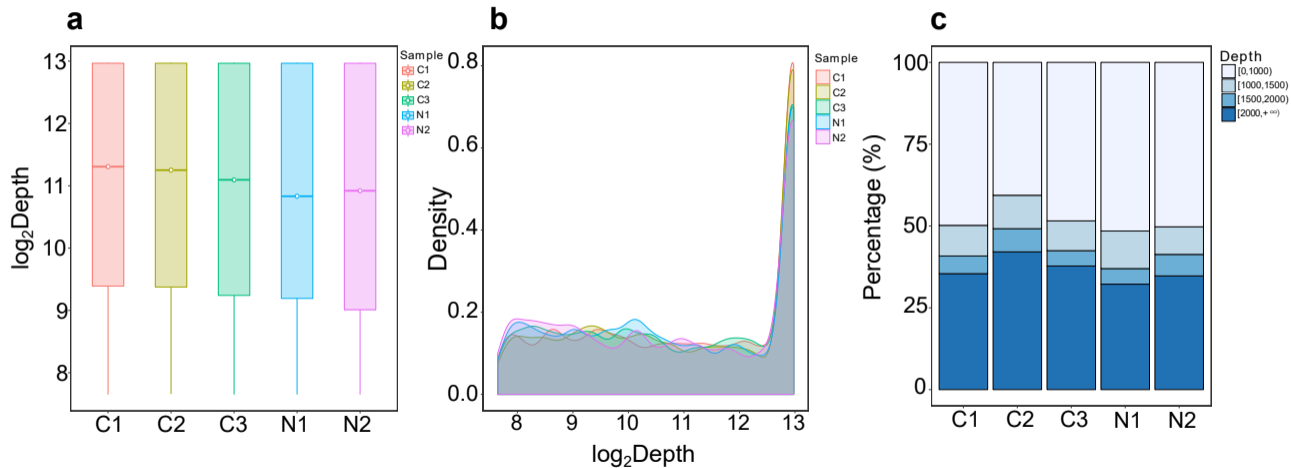

Fig. S7

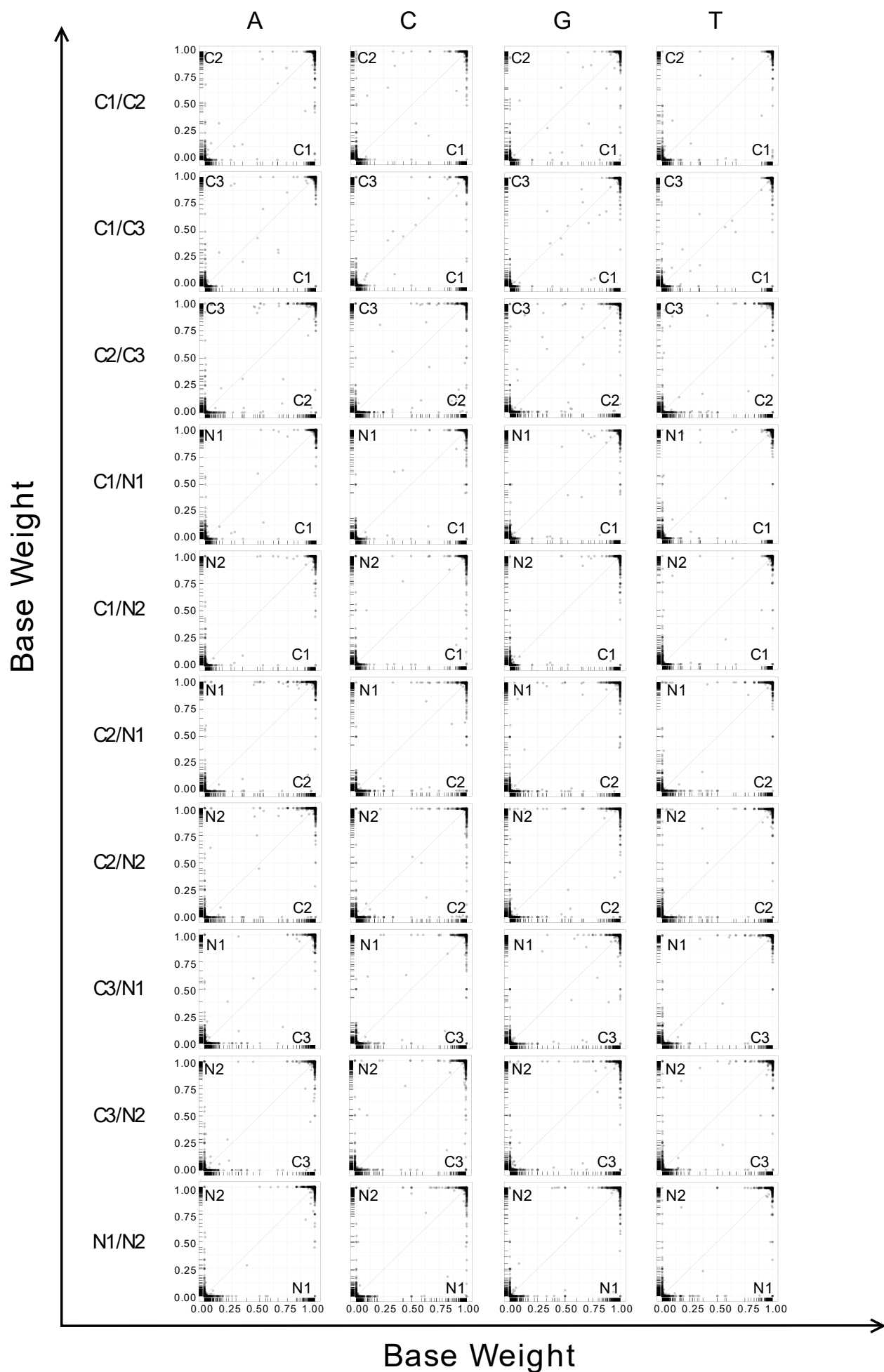

Fig. S8

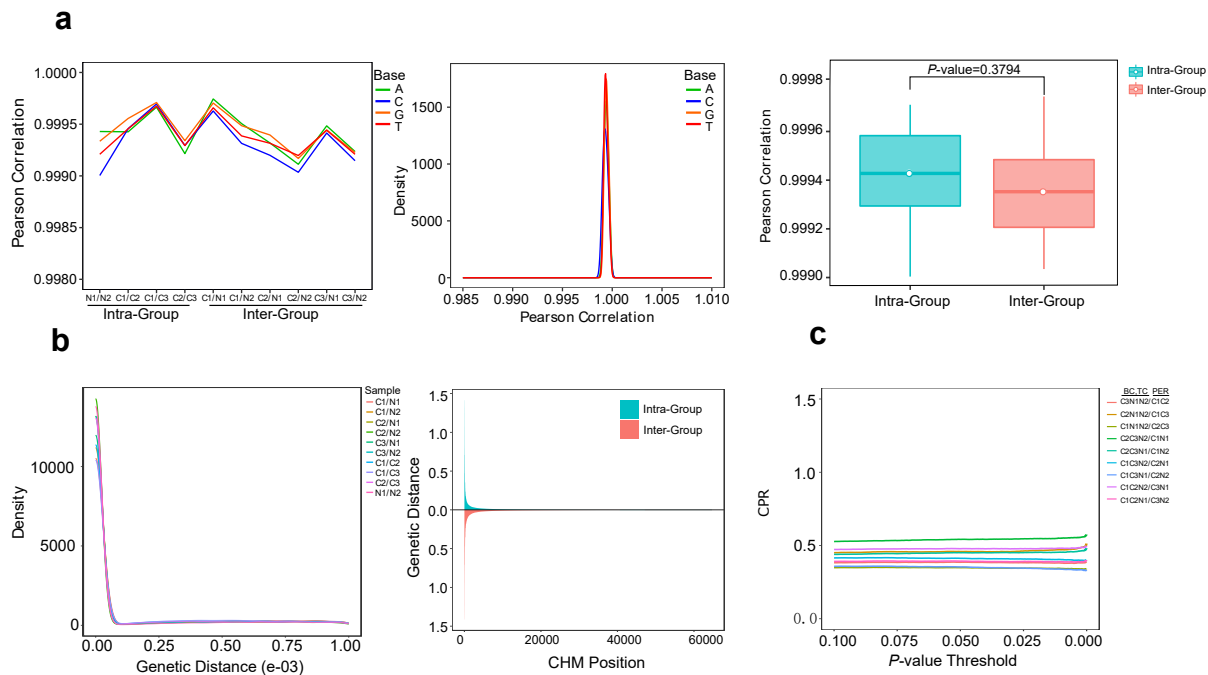

Fig. S9

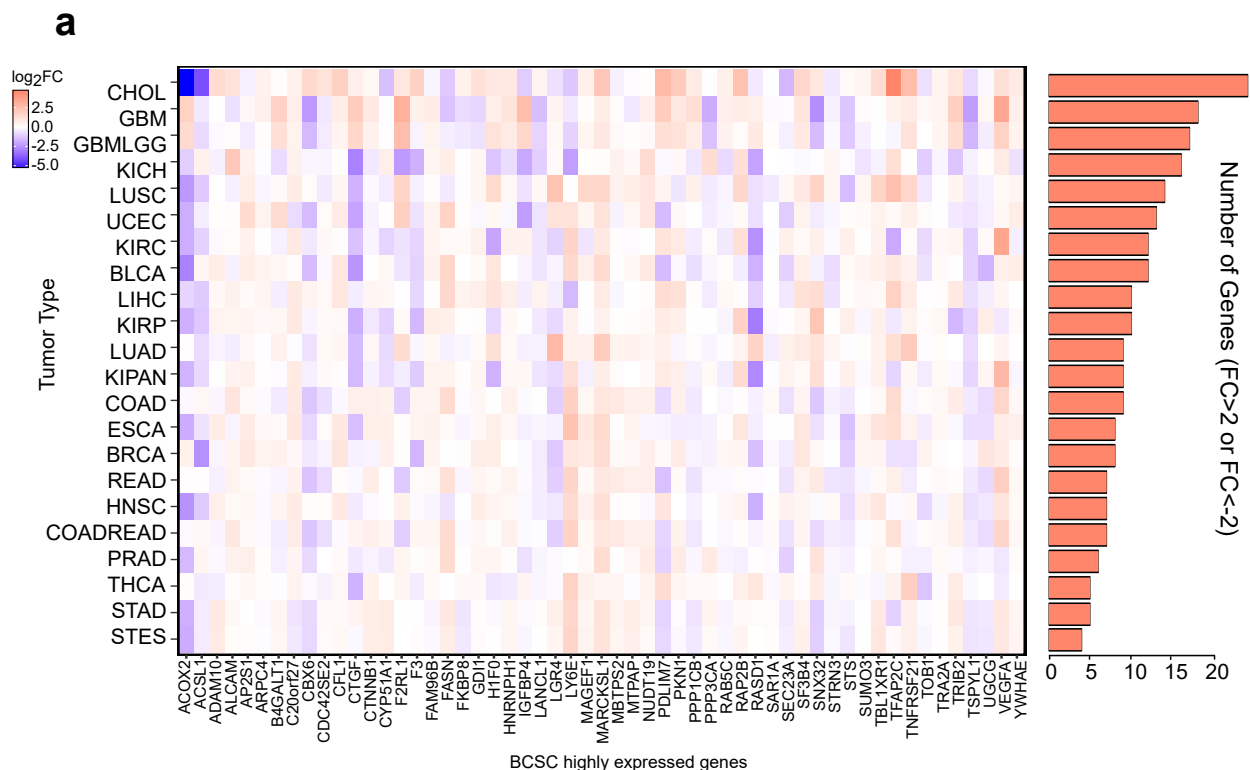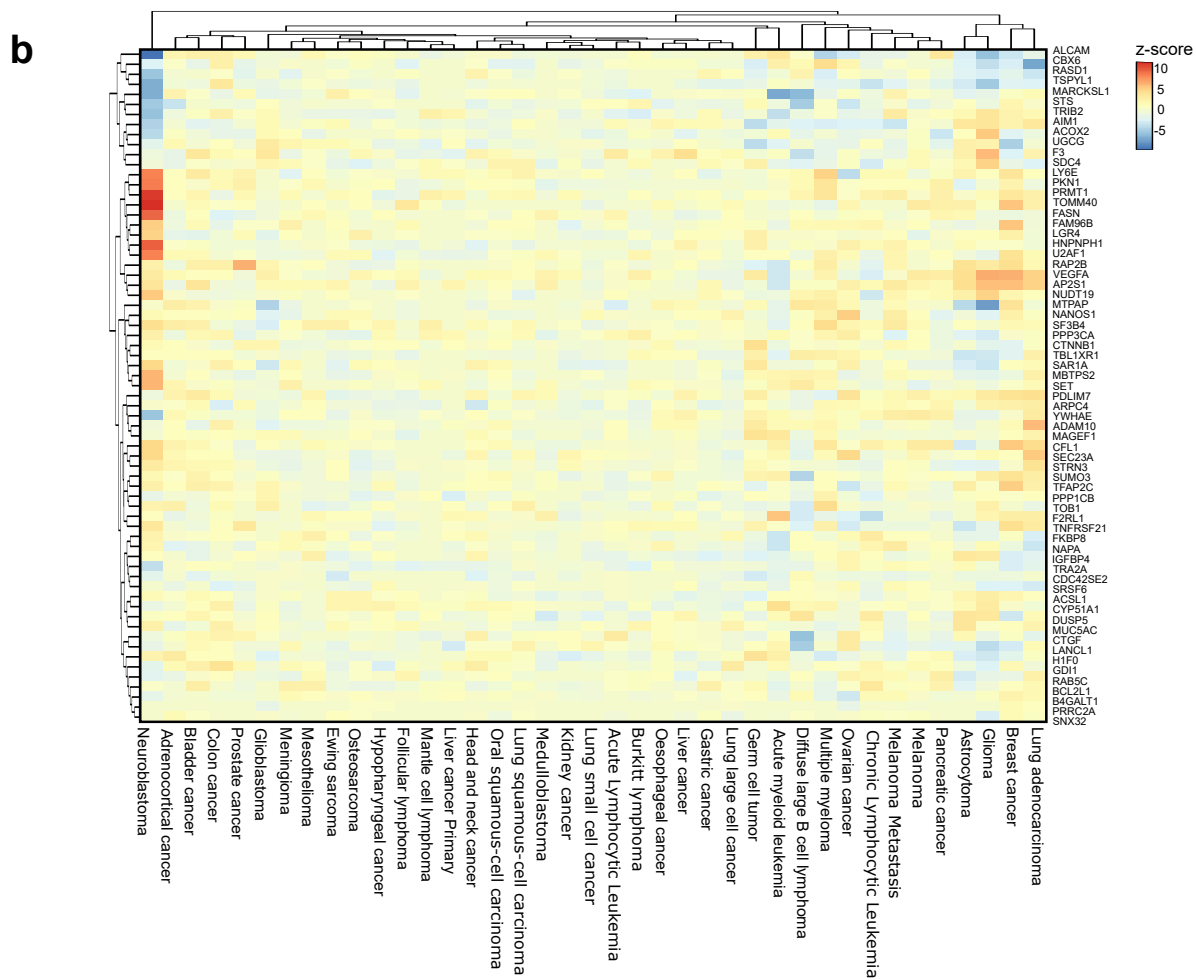

Relapse-free survival rate (%)

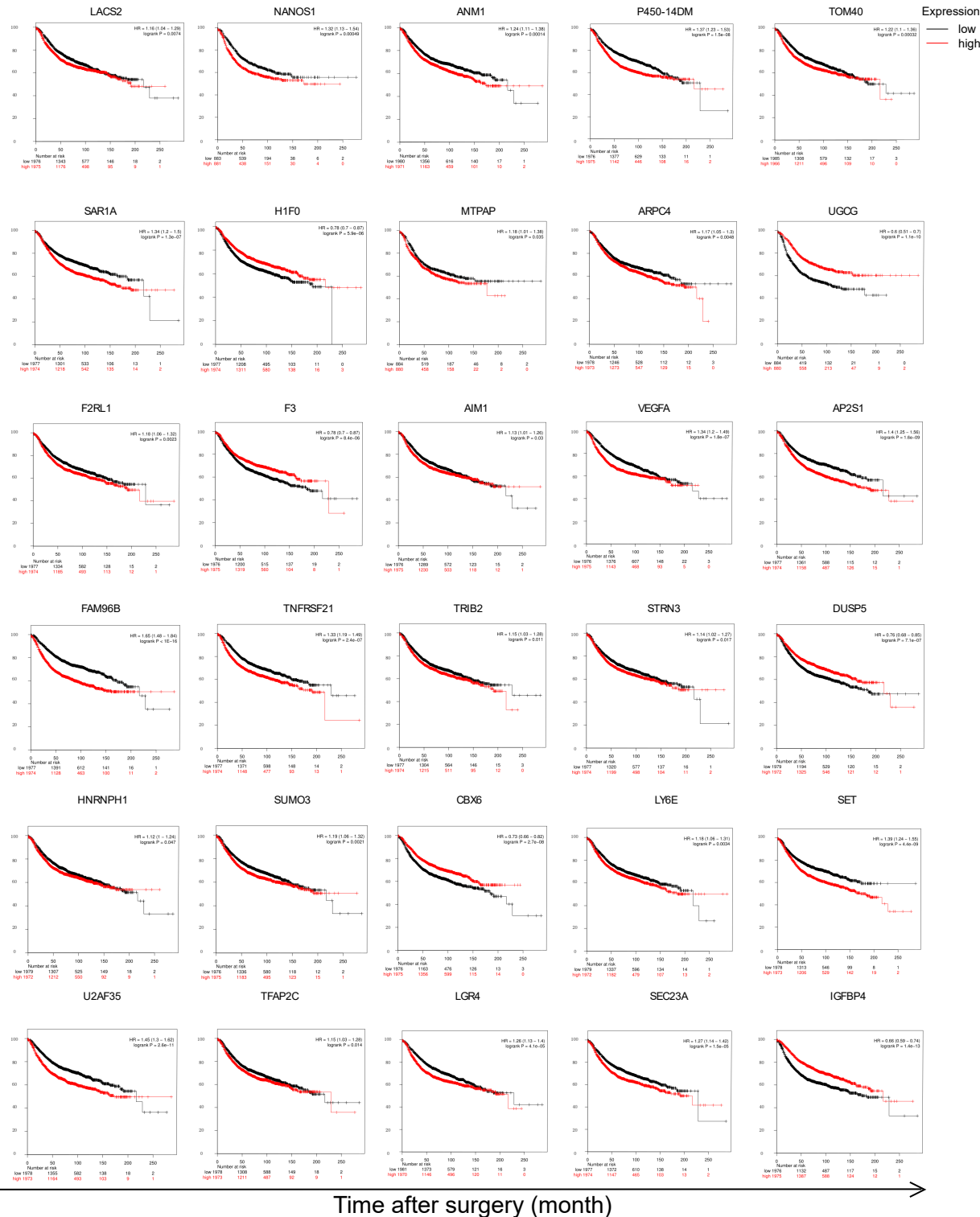

Fig. S11

Supplement: Supplementary file 7 — Additional file 7: Fig. S1. Potential mutation hotspots associated with breast cancer stem cells (BCSCs) are identified by bulk-cell whole-genome sequencing (WGS). a, Distances of potential single nucleotide variations (SNV) sites follow an exponential distribution. b, Two hotspots in chromosome 6 highlighted with a yellow bar are displayed as an example. c and d, Distribution of length (C) and proportion of functional annotations (D) for hotspots. Fig. S2. This figure related to Figure 1A. Serial sphere formation assay. a, Serial sphere formation assay from the first to fourth generation was performed in MDA-MB-231 cells. The spheres were photographed using an inverted microscope (Olympus). Scale bar, 200 μm. b, Cell number of spheres from the first to fourth generation. c, Expression levels of markers related to cancer stem cells [nanog homeobox (NANOG) and SRY (sex determining region Y)-box 2(SOX2)] was assessed by western blot assay in both enriched spheres (SP) and monolayer parental cells (2D). Fig. S3. Bulk-cell target deep DNA sequencing data evaluation. The violin plot (A) illustrates the distribution of depth in the target deep DNA sequencing, and the reads coverage distribution of each hotspot are shown by the pile-up bar plots (B). Fig. S4. Single-cell sphere formation assay. Images of single cell-derived spheres (red, BCSCs) and single cells that could not form spheres (green, non-BCSCs). The spheres and single cells were photographed using an inverted microscope (Olympus). Scale bar, 50 μm. Fig. S5. Data evaluation of single-cell target deep DNA sequencing of the hotspot region panel. a and b, Depth distribution of target deep DNA sequencing of hotspots from 5 samples. c and d, Reads coverage distribution of hotspots. Fig. S6. Pearson correlations of the genomic program (the hotspot region panel) between every two samples. Fig. S7. Data evaluation of single-cell target deep DNA sequencing of the cancer hotspot mutation (CHM) panel. a and b, Depth distr [file 40880_2018_326_MOESM7_ESM.pdf]
